# Increased genetic variation of A(H3N2) virus from influenza surveillance at the end of the 2016/2017 season for Shanghai port, China

**DOI:** 10.1038/s41598-022-19228-y

**Published:** 2022-10-12

**Authors:** Zilong Zhang, Shenwei Li, Xiaolin Zhu, Jian Hou, Hong Zhang, Baihui Zhao, Zhengan Tian

**Affiliations:** 1 Shanghai International Travel Healthcare Center, Shanghai, 200335 China; 2 Shanghai Customs District P.R.China, Shanghai, 200135 China; 3 Bio-X Life Science Research Center, Shanghai Jiao Tong University, Shanghai, 200030 China; 4 Shanghai BioGerm Medical Biotechnology Co., Ltd, Shanghai, 201401 China

**Keywords:** Viral genetics, Phylogenetics

## Abstract

Influenza A(H3N2) virus exhibited complex seasonal patterns to evade pre-existing antibodies, resulting in changes in the antigenicity of the viron surface protein hemagglutinin (HA). To monitor the currently imported influenza viruses as well as to assess the capacity of health emergencies at the Shanghai port, we collected respiratory specimens of passengers from different countries and regions including some of Europe with influenza-like illness at the Shanghai port during 2016/2017, examined amino acid substitutions, and calculated the perfect-match vaccine efficacy using the *p* epitope model. Phylogenetic analysis of the HA genes revealed that influenza A(H3N2) viruses belonging to eight subclades were detected, and three amino acid substitutions in the subclade 3C.2a.4 were also added. Besides, two epidemic influenza virus strains were found in the 2016/2017 winter and 2016 summer. The results of lower predicted vaccine effectiveness in summer suggest that the imported A(H3N2) strains were not a good match for the A/Hong Kong/4801/2014 vaccine strain since the summer of 2017. Therefore, the Shanghai Port might stop the risk of the international spread of influenza for the first time, and curb the entry of A(H3N2) from overseas at the earliest stage of a probable influenza pandemic.

## Introduction

Influenza viruses in the *Orthomyxoviridae* family cause highly contagious respiratory diseases with potentially fatal outcomes. There are currently four types in this family, which are type A, B, C and D. Type D viruses have not been reported to infect human yet^1^. In contrast to type B and C viruses, type A viruses in humans evolve fast and spark a devastating pandemic^2–4^. In particular, H3N2 subtype viruses hold responsible for a major seasonal influenza epidemic. Additionally, H3N2 subtype viruses could escape host immunity through piecemeal recombination, antigen drift or antigen conversion, and finally induce a lethal new flu pandemic with a potential to kill millions^5^.

On the surface of H3N2 subtype viruses, hemagglutinin (HA) proteins are closely related to the antigen variation of the epidemic influenza virus, and the variants further trigger phylogenetic clade changes. Since the spring and the summer of 2009, there have been up to seven clades (clades 1 to 7) defined by phylogeny inference^6^. After 2011, the derivative of Clade 3C, 3C1, 3C2, and 3C3 were dominated in many regions^7,8^. Previous studies demonstrated similar rates for clades 3C.2a, 3C.3, and 3C.3a early in the season^9^. In 2014, more new genetic subclades with special HA mutation sites emerged, 3C.2a from 3C2, 3C.3a and 3C.3b from 3C3^10^. Past research showed similar rates for clades 3C.2a, 3C.3, and 3C.3a early in the season, but 3C.2a dominated rapidly in the virus population for more than 70% by January 2015^7^. The genetic subclade 3C.2a1 emerged at the end of the 2015/2016 season^11^ and has become predominant in the 2016/2017 season. Afterwards, A(H3N2) 3C.2a further divided into new genetic groups by genetic drift (3C.2a.2, 3C.2a.3 and 3C.2a.4)^12–14^.

Compared to former years, the influenza season 2016/2017 co-circulated earlier in China, particularly in the southern regions. The influenza cases continued to rise, and A(H3N2) viruses became the dominant strain. Hong Kong SAR government continues to report a significant number of serious influenza-related cases and deaths. By June 11st 2017, the Hong Kong Center for Health Protection had confirmed 223 cases, with 155 deaths^15^. A(H3N2) viruses were also dominant in Europe and North America. The laboratory experiments verified that the vaccine effect of influenza A was not ideal for people over 65 from Finland^16^ and Sweden^17^. There were higher mortality and hospitalization rate in the United States in the 2016/2017 flu season^18^. Therefore, it is extremely vital to evaluate whether the vaccine matches the strains in circulation.

Shanghai is one of the port cities of China to the world, from which H3N2 subtype data might indicate the worldwide trend of the viruses’ evolution to some extent. Here, we analyzed virological surveillance data at the Shanghai Port, described the phylogenetic evolution, inspected the antigen variation characteristics from the molecular level of the currently circulating viruses, and compared them with the vaccine and WHO reference viruses representing various genetic clades. Our findings highlighted the structural implications for the understanding of the phenotypic changes, evolution, and epidemiological monitoring of A(H3N2) viruses.

## Results

### Virological influenza surveillance during flu season 2016/2017

Virological influenza surveillance data in the Shanghai port were collected weekly. From February 2016 to September 2017, a total of 64 swab samples were collected from passengers of different countries, including 41 passed through Asia (25 from Hong Kong and 12 from Southeast Asia, especially), 16 passed through Europe, 7 passed through the America, and 7 passed through Oceania.

A(H3N2) virus activity increased from the 44th week of 2016, peaked in the 1st week of 2017 and decreased afterwards. The highest proportion of A(H3N2) was observed in summer (28/64, 43.7%), followed by winter (22/64, 34.3%) which outnumbered by that in spring and fall (11/64, 21.8%).

### Phylogeny relationships of imported A(H3N2) viruses during the flu season 2016/2017

**Figure 1:**
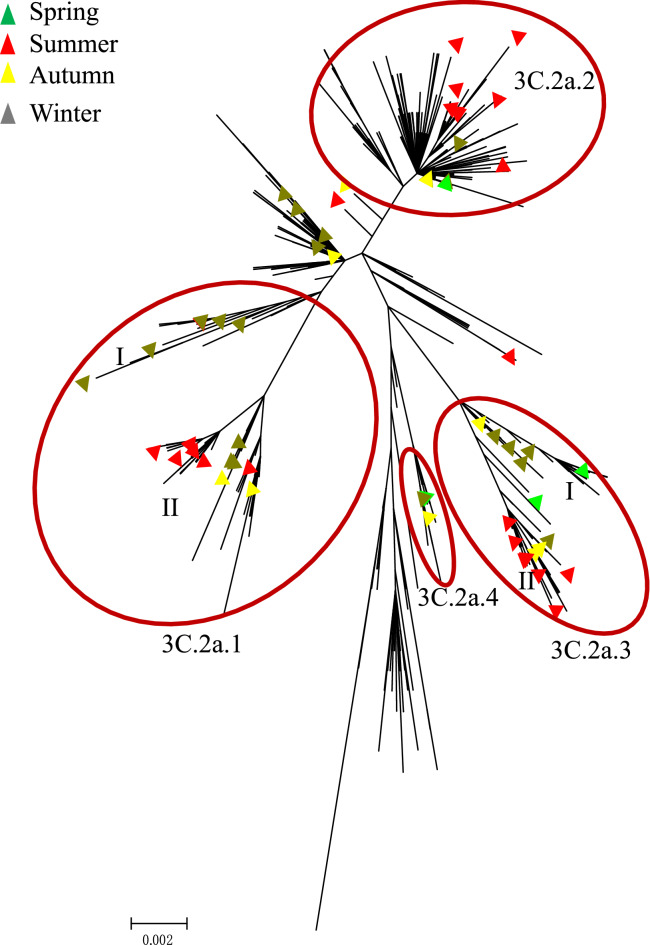
Phylogenetic analysis of the HA segments circulating between 2016/2017.

Of the 610 genetically characterized viruses, 546 were provided from GISAID EpiFlu databases. All 64 HA genes sequenced by the Shanghai Port belonged to the H3N2 3C.2a clade. This clade also included the vaccine strain A/HongKong/4801/2014, supporting the vaccine recommendation in the 2016–2018 northern hemisphere influenza season by WHO. Among the 64 viruses, the majority (n = 20, 31.2%) belonged to the subclade 3C.2a.1 represented by A/Singapore/INFIMH-16-0019/2016. The proportions for other subclades were 26.5% (3C.2a.2, n = 17), 25% (3C.2a.3, n = 16) and 6.2% (3C.2a.4, n = 4) (Fig. 1).

**Figure 2:**
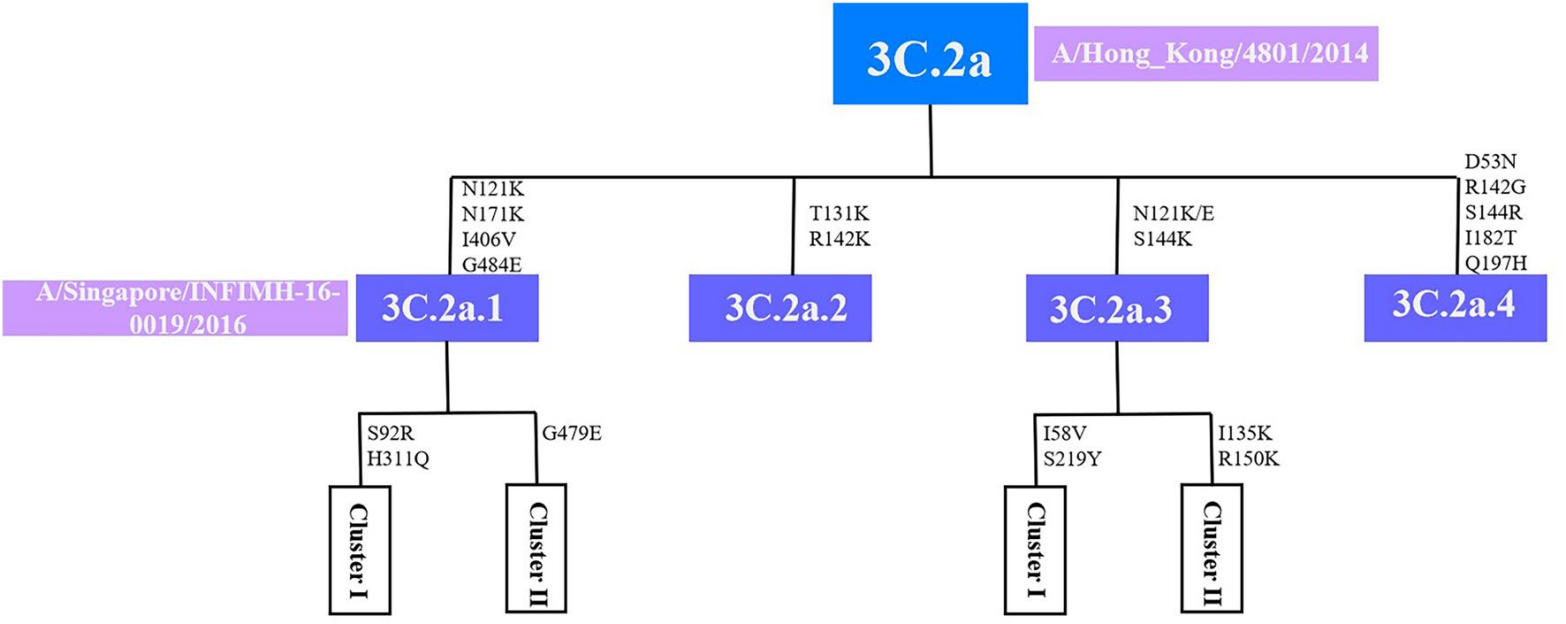
Schematic diagram demonstrating the shared amino acid changes between clades 3C.2a, 3C.2a.1, 3C.2a.2, 3C.2a.3 and 3C.2a.4 on HA gene.

Individual clades of A(H3N2) are typically defined by amino acid substitutions that occur as they diversify from parental strains. Analysis of HA sequences indicated co-circulation of multiple variants in clade 3C.2a. All variants within subclade 3C.2a.1 shared four substitutions N121K, N171K, I406V and G484E. Three additional substitutions were observed in the 3C.2a.1 subcluster: S92R and H311Q in cluster I, G479E in cluster II. Variants 3C.2a.3 shared N121K/E and S144K (I58V and S219R in cluster I and T135K and R150K in cluster II), Variants 3C.2a.2 were characterized by T131K and R142K substitutions and variants 3C.2a.4 were characterized by D53N, R142G, S144R, I182T and Q197H (Fig. 2).

There were more 3C.2a.1 variants identified from samples collected in the 2017 summer (n = 11) than in the 2016/2017 winter (n = 7). This subclade was further divided into two homogenous sub-clusters (cluster I and II; Fig. 1). The strains from cluster I were concentrated in winter, and the cluster II strains were persisted more common in the summer months. Most viruses in the subgroup 3C.2a.3 happened in summer. And we also found that there was no prominent summer or winter trend of viruses clustered in 3C.2a.2.

### The clade pattern of imported A(H3N2) influenza viruses, 2016/2017

**Figure 3:**
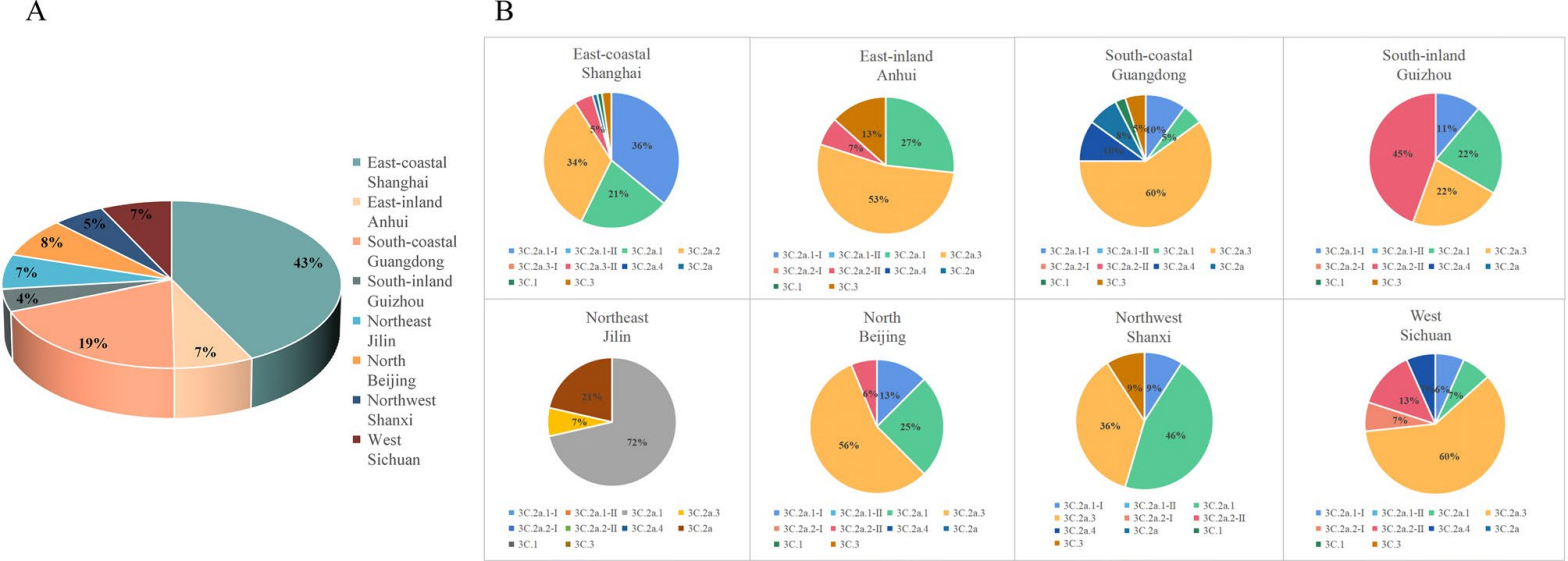
The Proportions of Influenza A(H3N2) in China six regions (**A**) and H3N2 clade patterns in China eight provinces (**B**).

To analyze the geographical distribution of A(H3N2) in China, 31 provinces were classified into six regions based on geographic proximity: North (Beijing), East-coastal (Shanghai), East-inland (Anhui), South-coastal (Guangdong), South-inland (Guizhou), Northeast (Jilin), Northwest (Shanxi) and West (Sichuan). According to our phylogenetic analysis, the A(H3N2) number of the above six regions be counted (Fig. 3A). The Proportions for A(H3N2) in these regions were 5%, 43%, 7.2%, 19%, 4%, 7% ,5% and 7%, respectively. Interestingly, higher epidemic waves of A(H3N2) were observed in Eastern and Southern in China coastal areas, and we presumed that convenient transportation and dense population contributed to it^19^.

The genetic diversity results (Fig. 3B) indicated that the diversity increased in the East and South, especially coastal cities, Shanghai and Guangzhou. All clades and subclades of the current A(H3N2) were detected in both cities. 3C.2a.3 (60%) was dominant in Guangzhou, with a small proportion of 3C.2a.4 (10%), 3C.2a (3%), 3C.1 (2%), 3C.3 (5%), 3C.2a.1-I (10%) and 3C.2a.1 (5%). In contrast, 3C.2a.1, 3C.2a.1-I and 3C.2a.2 were the major subcluster in Shanghai, with proportions of 19.21%, 32.36% and 30.34%, respectively. 3C.2a.3-II (4%), 3C.1 (1%), 3C.3 (2%) and 3C.2a (1%) were also detected in this region. The diversity of the clade pattern and the dominant clade in these two coastal cities matched well with the trends of the current global A (H3N2), likely because of the higher density of migration and subtropical monsoon climate^20^.

### Prediction of glycosylation sites in A(H3N2) viruses during flu season 2016/2017

There were two models of predicted glycosylation sites in the HA proteins of the A(H3N2) clade 3C.2a: 12 potential glycosylation sites (N^8^ST, N^22^GT, N^38^AT, N^45^SS, N^63^CT, N^126^WT, N^133^GT, N^158^YT, N^165^VT, N^246^ST, N^285^GS and N^483^GT) and 11 potential glycosylation sites (N^8^ST, N^22^GT, N^38^AT, N^45^SS, N^63^CT, N^126^WT, N^133^GT, N^158^YT, N^165^VT, N^246^ST and N^285^GS). All of virus strains detected at the Shanghai Port in the clade 3C.2a.1 had 11 potential glycosylation sites, and the rest in the other clades had 12 sites. Comparing to the vaccine strains 2016/2017 A/HongKong/4801/2014 (N^8^ST, N^22^GT, N^38^AT, N^45^SS, N^63^CT, N^126^WT, N^133^GT, N^165^VT, N^246^ST, N^285^GS and N^483^GT), the clade 3C.2a.1 virus did not have potential glycosylation site 483(N^483^GT), the viruses in the clade 3C.2a.2, the clade 3C.2a.3 and the clade 3C.2a.4 had the potential glycosylation site 158(N^158^YT).

### Estimation of vaccine efficacy for A(H3N2)

To assess the effect of the accumulated mutations in the HA1 domain on predicted vaccine efficacy in a given year, the *p* epitope method was used to evaluate how closely the vaccine strain resembles the imported strain (Table 1). Theoretically, when *p* epitope in the dominant epitope is higher than 0.19, the vaccine efficacy becomes negative^21,22^. For the 2016/2017 season, the average *p* epitope for all A(H3N2) strains was 0.090, which indicated the vaccine efficacy (VE) against those strains was 52.96% (E = 24.89% of 47%, *p* epitope = 0) of that of a perfect match with the vaccine strain. However, from the 2016/2017 winter to the 2017 autumn, the VE value fluctuated first and then decreased, with the highest value in spring (VE = 58.51%), the lowest value in autumn (VE = 58.51%), and the inflection point in summer (VE = 49.29%). These results suggest that the A(H3N2) strains circulating in 2017 were separated from the vaccine strain and effectively reduced the VE starting in the summer.

**Table 1:** Calculated vaccine efficacy using *P*_epitope_ model in dominant epitope A of influenza A(H3N2) circulating in Shanghai port during 2016/2017, winter, spring, summer and autumn.

Influenzaseason	Vaccine strain	Dominantepitope	Pepitope	Vaccine efficacy 47%	Vaccine efficacy 100%
2016/2017	A/Hong_Kong/4801/2014 2016/2017	A	0.090	24.89%	52.96%
Winter			0.082	26.5%	56.53%
Spring			0.078	27.5%	58.51%
Summer			0.096	23.17%	49.29%
Autumn			0.105	21.00%	44.68%

## Discussion

Human seasonal A(H3N2) virus epidemics in different zone have highly diverse patterns, especially in the northern hemisphere, where these patterns can exhibit semiannual or annual epidemic cycles. Moreover, HA segments of A(H3N2) viruses have undergone considerable genetic differentiation and evolved in seven genetic groups and multiple clades/subclades since 2009. It is the result of H3N2 viruses circulating via the network of temporally overlapping epidemics and high rates of migration, rather than local persistence. It has been suggested that the global persistence of A(H3N2) virus is the result of a migrating meta-population in which multiple different localities may seed seasonal epidemics in temperate regions in a given year^23^. Shanghai, the most developed and open city in China, attracts people around the world. So, the Shanghai Port has the characteristics of large passenger flow, high workload and wide international flight distribution. Around 44 million passengers enter and exit the Shanghai Port in 2017, accounting for 7.3 percent of the total number of people entering and exiting China's ports^24^. In addition, the specimens involved in this study were all from international travelers, who pass through cities with high population density or dense traffic, such as Europe, the United States, and Hong Kong, China. These cities or regions provide convenient channels for the mutation, transmission and spread of influenza^25^. Although surveillance for influenza at ports has been increasing, there have been few reports of interactions between ports and global epidemic trends. In this study, we performed genetic and evolutionary analyses for viruses obtained during 2016/2017 in order to investigate the evolution of the influenza virus during 2016/2017 at the Shanghai Port and predict the influenza A(H3N2) virus epidemic trends in the future.

The genetic and phylogenetic analyses in different countries and regions indicated that there has been a similar pattern existed among all of the evolutionary trends of A(H3N2) viruses discussed above. Previous research was reported by either ethnically homogenous (Taiwan^26^, Australian^27^ and Canada^28^) or the GISAID EpiFlu databases (isolated in Japan, Bangladesh, Australia, Thailand and the USA). In the current study, 31.2% viruses of the 3C.2a clade clustered in a subgroup carrying N121K, N171K and G484E. In Europe and Canada, the majority of A(H3N2) viruses also belonged to the subclade 3C.2a.1 during the same season^28,29^, whereas amino acid substitutions were used as clade markers just at N171K or N121K. Most of the rest viruses evolved away from the 3C.2a-A/Hong Kong/4801/2014-like clade by acquiring the genetic markers T131K, R142K and N121K/E, S144K which have been identified as being characteristic of clades 3C.2a.2 and 3C.2a.3. These markers were reported in most of other H3N2 influenza viruses isolated in the Denmark, Finland, Israel, Korean, Yokohama, Taiwan and the UK, in 2016/2017^30–36^. Additionally, clade 3C.2a.3 was further grouped into cluster I (carrying I58V and S219Y) and cluster II (T135K and R150K). The substitutions in cluster I has appeared in report from the UK, and in cluster II emerged in the Taiwan putative clades 3C.2a.3a, which were isolated from severe patients. Members of the subclade 3C.2a.4 were characterized by two amino acid substitutions at R142G and S144R. Three other variants (D53N, I182T and Q197H) were not reported previously, which is likely to increases the odds of implications including alterations of the antigenic epitopes and immune escape. These reports, together with our results, suggested that subclusters within clade 3C.2a and eight subclades have emerged and expanded during this recent influenza epidemic. These changes were continuing to diversify worldwide with complicated and rapid dynamics.

During 2016/2017, the following two influenza epidemics occurred in this study: one epidemic between December 2016 and February 2017 and one epidemic between June 2017 and August 2017. These epidemics were consistent with the influenza epidemics observed in Hong Kong, Southeast Asia and Europe in 2017. According to the previous research on influenza seasons, Influenza cases usually appear between autumn and spring, with the influenza activity peaking after October^37,38^. However, the A(H3N2) virus seemed to frequently result in larger summer epidemics, such as those in 2010 and 2012^39,40^. In our study, the H3N2-dominant summer wave occurred in 2017. The number of 2017 summer season was over than the 2016/2017 winter season. Research has shown that the I668V mutation in the PA subunit led to temperature-sensitive and attenuation in the 2016/2017 winter season virus strains and an adaptation to high temperatures in the 2017 summer viruses^41^. Adaptation to high temperatures may be the result of the natural evolution of the influenza virus and could explain the second epidemic that occurred during the summer of 2017 in this study.

Furthermore, we predicted the VE of the 2016/2017 season viruses by the *p* epitope model and found that predicted VE value to decline overall in 2016/2017. From 2016, A/Hong Kong/4801/2014 is regarded as the vaccine strain in the southern hemisphere, and was well-matched for the imported A(H3N2). Early and mid-season VE estimates for 2016/2017 showed that the A(H3N2) illness had observed antigenic drift and decreased predicted VE^29^. But in the 2016/2017, it was successively reported that the imported A(H3N2) strains had observed antigenic drift and decreasing predicted VE. Denmark and Finland’s studies indicated a drop in VE from the early period to the later period of the 2016/2017^42^. Coincidentally, this phenomenon exists in 2017/2018 too. In USA analysis of 2017/2018 of A(H3N2) VE in prevention of hospitalization, VE was low across adult age groups and levels of frailty and chronic comorbidities^43^. We furthermore found that the main antigenic change is the substitution of amino acid 135, 150 and 131 in mid to late 2017 (Table S1). Amino acid 135 is located in a conserved region of the receptor-binding site in the antigenic epitope A and causes a loss of a glycosylation motif^44^. Changes in glycosylation motifs may be relevant to antigenicity, viral fitness and/or pathogenicity^45^. Amino acid 131 is located in the antigenic epitope A and conserved in 45% of all human H1, H2 and H3 viruses^46^. VE studies from Stockholm and Finland show that the proportion of samples containing T131K (36%) increased may be responsible for viral antigenic change and part of the observed VE drop^33^. Studies from northern Greece have provided evidence supporting indications that the specific T135K variant may be associated with vaccination failure. The T135K mutation was observed in 82% of the viruses originating from vaccinated patients^44^. Based on the Canadian report, the higher VE estimates may be due to the relatively infrequent (15%) circulation of the T135K variant in Canada^47^. T135K-R150K are also as genetic markers for clade 3C.2a.3, and viruses in this clade were isolated from patients with severe infections in Taiwan. This finding can be explained by the lower VE and shorter protection time for these clades compared with other circulating clades^48^. As a result, decreasing influenza vaccine protection with increasing time, A/Hong Kong/4801/2014 may not be as effective in eliciting immunity against future circulating A(H3N2) in the next influenza season. If the emerging A(H3N2) virus subpopulation continues to diversify from vaccine components, VE may decrease further by the end of the 2016/2017 season in terms of its antigenic properties or a broader cycle of variant strains.

In addition, compared to A/Hong Kong/4801/2014, the 3C.2a strains in our surveillance data had at least eleven potential N-glycosylation sites and had similar glycosylation patterns. Clade 3C.2a.1-like strains lost one potential glycosylation sites: 483(N^483^GT), Clade 3C.2a.2-like, 3C.2a.3-like, and 3C.2a.4-like strains gained one potential glycosylation site: 158(N^158^YT). Research shows that the mechanisms of glycosylation and deglycosylation in virus fall into three general classes: (i) mask antigenic epitopes and thus block binding to neutralizing antibodies^49^, (ii) adjust receptor-binding affinity^50^, or (iii) modulate the virulence of the Influenza viruses in mammals^51^. It’s could be concluded that the virus would make itself to elude the existed antibodies by changing herd immunity or increasing the viral fitness in an unknown mechanism.

In summary, our study indicates that new mutations and derivations of clade 3C.2a emerged continuously and rapidly during 2016/2017, which can reflect the trend of the current global influenza A(H3N2), and also has a paramount impact on the viral adaptation and transmission. So Shanghai might stop the risk of the international spread of influenza for the first time, and curbing the entry of A(H3N2) from overseas at the earliest stage of the influenza pandemic. Strengthening the entry monitoring of ports in coastal areas and improving the ability of ports health quarantine to deal with public health emergencis are required to investigate the antigenic effects and to avoid the next pandemic.

## Methods

### Ethics statements

This study was performed with the residual samples collected for the detection of influenza in the Shanghai Port. All the samples used for research have got the informed consent of passengers. The sample for the informed consent was attached as follows.

The study protocols were approved by the Institutional Review Board of Bio-X Life Science Research Center of Shanghai Jiao Tong University (IRB No. M202007). Informed consent was obtained from all subjects, and all methods were carried out in accordance with relevant guidelines and regulations.

### Surveillance and data collection

The Shanghai port located on the south wing of the Yangtze River Delta Region. It is a key transportation hub combing water, land, and air transportation, facilitating the airborne survival and transmission of influenza viruses. During 2016/2017, a total of 64 throat swabs were collected from influenza-like illness cases in the Shanghai port. The basics epidemiological data including patients’ age, stopover sites and sampling dates were collected. The detailed list of viruses is provided in Table 2.

**Table 2:** Influenza A(H3N2) virus strains sequenced in this study.

Name	Patients’age	Nationality	Stopover sites	Sampling dates	Accession numbers
201602161010.seq	9	China	Hong Kong	2016.02.16	OM956282
201608081702.seq	29	China	Australia, Hong Kong	2016.08.08	OM956281
201609171121.seq	29	China	Hong Kong, Australia	2016.09.17	OM956280
201610051316.seq	31	China	Hong Kong	2016.10.05	OM956279
201610241504.seq	44	China(Taiwan)	Japan, the USA, Hong Kong, Taiwan	2016.10.24	OM956257
201610251519.seq	24	China	Hong Kong	2016.10.25	OM956278
201611301787.seq	25	China	Hong Kong	2016.11.30	OM956277
201612141923.seq	2	China	Hong Kong	2016.12.14	OM956276
201612261085.seq	20	China	Hong Kong	2016.12.26	OM956275
201701051269.seq	49	Australia	Italy	2017.01.05	OM956256
201701071301.seq	63	The Netherlands	The Netherlands	2017.01.07	OM956255
201701111341.seq	65	China	Indonesia	2017.01.11	OM956228
201701121362.seq	23	China	The United Arab Emirates(UAE)	2017.01.12	OM956227
201701121363.seq	62	China	Vietnam	2017.01.12	OM956274
201701151395.seq	38	China	The USA	2017.01.15	OM956254
201701211465.seq	13	China	Britain	2017.01.21	OM956253
201701211466.seq	53	China	France	2017.01.21	OM956252
201701241505.seq	3	China	Italy	2017.01.24	OM956273
201701261555.seq	36	Germany	Germany	2017.01.26	OM956251
201701291611.seq	30	China	Macao	2017.01.29	OM956225
201702051704.seq	10	China	The USA, Hawaii	2017.02.06	OM956231
201702101743.seq	58	China	Iceland, Norway	2017.02.10	OM956250
201702121780.seq	34	China	Canada	2017.02.12	OM956249
201702131787.seq	1	The USA	The USA	2017.02.13	OM956248
201702211871.seq	45	China	Canada	2017.02.22	OM956247
201702211874.seq	23	Indonesia	Indonesia	2017.02.21	OM956258
201702221882.seq	64	Netherlands	Netherlands, France	2017.02.22	OM956246
201702261906.seq	30	China	The Netherlands	2017.02.26	OM956245
201703041965.seq	30	China	Germany, South Korea	2017.03.06	OM956244
201703081006.seq	55	Russia	Russia, Indonesia	2017.03.08	OM956243
201705111650.seq	32	Indonesia	Indonesia, Malaysia	2017.05.11	OM956224
201705191715.seq	33	China	Hong Kong	2017.05.19	OM956272
201706081022.seq	67	China	Turkey	2017.06.08	OM956242
201707231849.seq	12	China	Hong Kong	2017.07.23	OM956283
201707231857.seq	59	China	Hong Kong	2017.07.23	OM956271
201707241890.seq	54	China	Hong Kong, Thailand	2017.07.24	OM956270
201707261936.seq	69	China	Hong Kong	2017.07.26	OM956269
201707271994.seq	21	China	Macao, Hong Kong	2017.07.27	OM956268
201707271996.seq	54	China	Hong Kong	2017.07.27	OM956267
201707301041.seq	8	China	Indonesia	2017.07.30	OM956230
201707301044.seq	72	China	Russia	2017.07.30	OM956241
201708061243.seq	21	China	Japan	2017.08.06	OM956266
201708061245.seq	26	China	The USA, Canada	2017.08.07	OM956240
201708071293.seq	38	China	Hong Kong, Thailand	2017.08.07	OM956265
201708101387.seq	20	China	Hong Kong	2017.08.10	OM956264
201708111416.seq	71	China	France, Switzerland, Italy	2017.08.11	OM956239
201708111419.seq	9	China	Hong Kong, Macao	2017.08.11	OM956263
201708121441.seq	5	China	Hong Kong	2017.08.12	OM956262
201708131464.seq	24	China	South Korea	2017.08.13	OM956226
201708151516.seq	76	China	Russia	2017.08.15	OM956238
201708191682.seq	16	China	South Korea	2017.08.19	OM956223
201708221788.seq	18	China	Germany, France, Switzerland, Italy	2017.08.22	OM956237
201708241888.seq	5	China	Taiwan	2017.08.24	OM956221
201708251901.seq	24	China	Philippines, Hong Kong	2017.08.25	OM956220
201708251923.seq	8	China	France, Italy, Turkey	2017.08.25	OM956236
201708261954.seq	22	China	Hong Kong, Thailand	2017.08.26	OM956261
201708261955.seq	19	China	Hong Kong	2017.08.26	OM956260
201708281998.seq	62	China	Russia	2017.08.28	OM956235
201708301087.seq	18	China	Taiwan	2017.08.30	OM956229
201709041192.seq	34	China	Hong Kong, Australia	2017.09.04	OM956259
201709131430.seq	46	Poland	Poland	2017.09.13	OM956232
201709231691.seq	30	South Korea	Indonesia, South Korea	2017.09.25	OM956222
201709261774.seq	33	India	Hong Kong, India	2017.09.26	OM956234
201709301835.seq	25	China	Hong Kong	2017.10.03	OM956233

### RNA purification and HA gene sequencing

Viral RNA was obtained from 140 μL of the sample by the QIAMP viral RNA extraction kit (Qiagen, Hilden, Germany) according to the manufacturer's instructions. RNA was eluted in 100 μl RNase/DNase elution buffer provided in the kit and stored at − 40 °C. Samples were subtyped based the Real-Time RT-PCR Diagnostic assay (Shanghai Biogerm Influenza H1/H3 typing assay) for detection of influenza virus in an ABI Prism 7500 thermocycler (Applied Biosystems, Foster City, California, USA). The HA genes were subsequently amplified by the samples with a Ct value of less than 30, and were subjected to reverse transcription and amplification using a PrimeScript One-step RT-PCR Kit Ver.2 (TaKaRa, Japan) as previously described^52^. RT-PCR products with the correct band size were selected by agarose gel electrophoresis and sequenced using ABI PRISM Dye Deoxy Terminator cycle sequencing kit (Life Technology, Foster City, CA, USA). These HA gene sequences were assembled using the SeqMan Pro software (DNASTAR, Madison, WI) and deposited in the National Center for Biotechnology Information (https://www.ncbi.nlm.nih.gov/) with the accession numbers (OM956220-OM956283). Finally we obtained 64 full-length HA sequences.

### Sequence alignment and phylogenetic analysis

Reference sequences of known clades and vaccine strains (the accession numbers in Supplementary Table S2) as recommended by WHO^53^ included in the evolutionary analysis were retrieved from GISAID EpiFlu databases. All HA gene sequences aligned by MAFFT program (MAFFT-7.220-WIN64 version). Protein translation were performed on the basis of nucleotide sequences using Molecular Evolutionary Genetics Analysis software (MEGA, version 6.0; http://www.megasoftware.net/). Phylogenetic tree was constructed using the maximum-likelihood method with a Hasegawa-Kishino-Yano (HKY) + gamma nucleotide substitution model and 1000 bootstrap replications. The ability to perform clade designations based on signature amino acids as compared to A/HongKong/4801/2014-likeA/H3N2-like clade 3C.2a viruses and A/Singapore/INFIMH-16-0019/2016-likeA/H3N2-like clade 3C.2a.1 viruses was confirmed with the 64 isolates depicted in the final tree, and extended to the other 546 isolates.

### Prediction of glycosylation sites

The prediction of potential N-liked glycosylation sites was performed with an online server: NetNGlyc 1.0^54^. This server considers the amino acid alignment N-X-S/T, where X can be any amino acid except Asp or Pro. A threshold value of > 0.5 suggests glycosylation.

### Prediction of vaccine efficacy

The predicted VE of A(H3N2) was estimated using the Pepitope model, which is a measure of the antigenic distance between vaccine and circulating strains. Antigenic distance was calculated from the fraction of substituted amino acid residues in the dominant HA epitope^21^. The amino acid residues in the HA epitopes of A(H3N2) were pre-defined in the *p* epitope calculator to respectively possess 19, 21, 27, 41 and 22 amino acids^55^. The mathematical formula linking VE and the *p* epitope is given by VE = −2.47 × *p* epitope + 0.47 in which VE is 47% when *p* epitope = 0.

## Supplementary Information

Supplementary Tables.

## Data Availability

The datasets generated and analysed during the current study are available in the National Center for Biotechnology Information (https://www.ncbi.nlm.nih.gov/), ACCESSION NUMBER TO DATASETS: OM956220-OM956283.
