# Supplementary material for: Increased genetic variation of A(H3N2) virus from influenza surveillance at the end of the 2016/2017 season for Shanghai port, China
Source: Sci Rep. 2022 Oct 12;12:17089. doi: 10.1038/s41598-022-19228-y (PMC9556717; doi:10.1038/s41598-022-19228-y)
Supplement: Supplementary file 1 — Supplementary Tables. [file 41598_2022_19228_MOESM1_ESM.docx]

**Title**

Increased genetic variation of human influenza A(H3N2) virus from influenza surveillance at the end of the 2016/17 season for people of entry to Shanghai

**Authors and Affiliations**

Zhang-zilong^1,2,#^ ,Li-shenwei^1,2,#^,Zhu-xiaolin^4^**^,#^**,Chu-yiwen^2^,Zhang-hong^1,2^,Zhao-baihui^3,4^,Tian-zhengan^1,2*^

^1^ Shanghai International Travel Healthcare Center, Shanghai 200335, China

^2^Shanghai Customs District P.R.China , Shanghai 200135, China

^3^Bio-X Life Science Research Center, Shanghai Jiao Tong University, Shanghai, China, 200030

^4^Shanghai BioGerm Medical Biotechnology Co.,Ltd, Shanghai 200135, China, 200120

^#^These authors contributed equally to this work.

* Corresponding author: Dr. Tian-zhengan

*Corresponding author email: tianzhenganciq@163.com

**Supplementary Table S1. Calculated vaccine efficacy using Pepitope model and number of mutations in dominant epitope of influenza A(H3N2) circulating in the Shanghai Port during the four seasons of 2016\17.**

| **Influenzaseason** | **Vaccine strain** | **Dominant epitope** | **No.of mutations** | **Residue differences** | **Pepitope** | **Vaccine efficacy 47%** | **Vaccine efficacy 100%** |
| --- | --- | --- | --- | --- | --- | --- | --- |
| **Winter** | A/Hong_Kong/4801/2014 2016/2017 | A | 1 | 135 | 0.053 | 34.00% | 72.34% |
|  |  |  | 1 | 140 | 0.053 | 34.00% | 72.34% |
|  |  |  | 1 | 144 | 0.053 | 34.00% | 72.34% |
|  |  |  | 2 | 122+144 | 0.105 | 21.00% | 44.68% |
|  |  |  | 2 | 131+144 | 0.105 | 21.00% | 44.68% |
|  |  |  | 4 | 135+142+144+150 | 0.211 | -5.00% | -10.64% |
| **Spring** |  |  | 1 | 142 | 0.053 | 34.00% | 72.34% |
|  |  |  | 2 | 142+144 | 0.105 | 21.00% | 44.68% |
|  |  |  | 2 | 142+144 | 0.105 | 21.00% | 44.68% |
|  |  |  | 1 | 144 | 0.053 | 34.00% | 72.34% |
| **Summer** |  |  | 1 | 135 | 0.053 | 34.00% | 72.34% |
|  |  |  | 1 | 142 | 0.053 | 34.00% | 72.34% |
|  |  |  | 2 | 131+135 | 0.105 | 21.00% | 44.68% |
|  |  |  | 2 | 131+142 | 0.105 | 21.00% | 44.68% |
|  |  |  | 3 | 135+144+150 | 0.158 | 8.00% | -10.62% |
|  |  |  | 4 | 135+142+144+150 | 0.211 | -5.00% | 72.36% |
| **Autumn** |  |  | 1 | 131 | 0.053 | 34.00% | 72.34% |
|  |  |  | 1 | 144 | 0.053 | 34.00% | 72.34% |
|  |  |  | 2 | 131+142 | 0.105 | 21.00% | 44.68% |
|  |  |  | 2 | 135+142 | 0.105 | 21.00% | 44.68% |
|  |  |  | 2 | 142+144 | 0.105 | 21.00% | -10.62% |
|  |  |  | 3 | 135+144+150 | 0.158 | 8.00% | 72.36% |

**Supplementary Table S2. Accession numbers in GISAID of HA influenza A(H3N2) and gene sequences used for phylogenetic tree analysis.**

| **Isolate_Name** | **Location** | **HA Segment_Id** | **Isolate_Id** |
| --- | --- | --- | --- |
| A/Liaoning-Yinzhou/1399/2017 | Asia / China | EPI1197750 \| 3000820918_N8K9F5ZU_v1_4 | EPI_ISL_303159 |
| A/Fujian-Licheng/1729/2017 | Asia / China | EPI1197726 \| 3000820919_N8K9F5ZZ_v1_4 | EPI_ISL_303156 |
| A/Zhejiang-Nanhu/1745/2017 | Asia / China | EPI1197710 \| 3000820917_N8K9F5ZP_v1_4 | EPI_ISL_303154 |
| A/Shanghai/9/2017 | Asia / China | EPI1194029 \| A/Shanghai/9/2017 | EPI_ISL_302580 |
| A/Shanghai/8/2016 | Asia / China | EPI1194021 \| A/Shanghai/8/2016 | EPI_ISL_302579 |
| A/Shanghai/7/2016 | Asia / China | EPI1194013 \| A/Shanghai/7/2016 | EPI_ISL_302578 |
| A/Shanghai/6/2016 | Asia / China | EPI1194005 \| A/Shanghai/6/2016 | EPI_ISL_302577 |
| A/Shanghai/53/2017 | Asia / China | EPI1193997 \| A/Shanghai/53/2017 | EPI_ISL_302576 |
| A/Shanghai/52/2017 | Asia / China | EPI1193989 \| A/Shanghai/52/2017 | EPI_ISL_302575 |
| A/Shanghai/51/2017 | Asia / China | EPI1193981 \| A/Shanghai/51/2017 | EPI_ISL_302574 |
| A/Shanghai/50/2017 | Asia / China | EPI1193973 \| A/Shanghai/50/2017 | EPI_ISL_302573 |
| A/Shanghai/5/2016 | Asia / China | EPI1193965 \| A/Shanghai/5/2016 | EPI_ISL_302572 |
| A/Shanghai/49/2017 | Asia / China | EPI1193957 \| A/Shanghai/49/2017 | EPI_ISL_302571 |
| A/Shanghai/48/2017 | Asia / China | EPI1193949 \| A/Shanghai/48/2017 | EPI_ISL_302570 |
| A/Shanghai/47/2017 | Asia / China | EPI1193941 \| A/Shanghai/47/2017 | EPI_ISL_302569 |
| A/Shanghai/46/2017 | Asia / China | EPI1193933 \| A/Shanghai/46/2017 | EPI_ISL_302568 |
| A/Shanghai/45/2017 | Asia / China | EPI1193925 \| A/Shanghai/45/2017 | EPI_ISL_302567 |
| A/Shanghai/44/2017 | Asia / China | EPI1193917 \| A/Shanghai/44/2017 | EPI_ISL_302566 |
| A/Shanghai/43/2017 | Asia / China | EPI1193909 \| A/Shanghai/43/2017 | EPI_ISL_302565 |
| A/Shanghai/42/2017 | Asia / China | EPI1193901 \| A/Shanghai/42/2017 | EPI_ISL_302564 |
| A/Shanghai/41/2017 | Asia / China | EPI1193893 \| A/Shanghai/41/2017 | EPI_ISL_302563 |
| A/Shanghai/40/2017 | Asia / China | EPI1193884 \| A/Shanghai/40/2017 | EPI_ISL_302562 |
| A/Shanghai/4/2016 | Asia / China | EPI1193876 \| A/Shanghai/4/2016 | EPI_ISL_302561 |
| A/Shanghai/39/2017 | Asia / China | EPI1193868 \| A/Shanghai/39/2017 | EPI_ISL_302560 |
| A/Shanghai/38/2017 | Asia / China | EPI1193859 \| A/Shanghai/38/2017 | EPI_ISL_302558 |
| A/Shanghai/37/2017 | Asia / China | EPI1193852 \| A/Shanghai/37/2017 | EPI_ISL_302557 |
| A/Shanghai/36/2017 | Asia / China | EPI1193844 \| A/Shanghai/36/2017 | EPI_ISL_302556 |
| A/Shanghai/35/2017 | Asia / China | EPI1193836 \| A/Shanghai/35/2017 | EPI_ISL_302555 |
| A/Shanghai/34/2017 | Asia / China | EPI1193828 \| A/Shanghai/34/2017 | EPI_ISL_302554 |
| A/Shanghai/33/2017 | Asia / China | EPI1193820 \| A/Shanghai/33/2017 | EPI_ISL_302553 |
| A/Shanghai/32/2017 | Asia / China | EPI1193812 \| A/Shanghai/32/2017 | EPI_ISL_302552 |
| A/Shanghai/31/2017 | Asia / China | EPI1193804 \| A/Shanghai/31/2017 | EPI_ISL_302551 |
| A/Shanghai/30/2017 | Asia / China | EPI1193796 \| A/Shanghai/30/2017 | EPI_ISL_302550 |
| A/Shanghai/3/2016 | Asia / China | EPI1193788 \| A/Shanghai/3/2016 | EPI_ISL_302549 |
| A/Shanghai/29/2017 | Asia / China | EPI1193780 \| A/Shanghai/29/2017 | EPI_ISL_302548 |
| A/Shanghai/21/2017 | Asia / China | EPI1193772 \| A/Shanghai/21/2017 | EPI_ISL_302547 |
| A/Shanghai/20/2017 | Asia / China | EPI1193764 \| A/Shanghai/20/2017 | EPI_ISL_302546 |
| A/Shanghai/2/2016 | Asia / China | EPI1193756 \| A/Shanghai/2/2016 | EPI_ISL_302545 |
| A/Shanghai/19/2017 | Asia / China | EPI1193748 \| A/Shanghai/19/2017 | EPI_ISL_302544 |
| A/Shanghai/18/2017 | Asia / China | EPI1193739 \| A/Shanghai/18/2017 | EPI_ISL_302543 |
| A/Shanghai/17/2017 | Asia / China | EPI1193731 \| A/Shanghai/17/2017 | EPI_ISL_302542 |
| A/Shanghai/16/2017 | Asia / China | EPI1193723 \| A/Shanghai/16/2017 | EPI_ISL_302541 |
| A/Shanghai/15/2017 | Asia / China | EPI1193715 \| A/Shanghai/15/2017 | EPI_ISL_302540 |
| A/Shanghai/14/2017 | Asia / China | EPI1193707 \| A/Shanghai/14/2017 | EPI_ISL_302539 |
| A/Shanghai/13/2017 | Asia / China | EPI1193699 \| A/Shanghai/13/2017 | EPI_ISL_302538 |
| A/Shanghai/12/2017 | Asia / China | EPI1193691 \| A/Shanghai/12/2017 | EPI_ISL_302537 |
| A/Shanghai/11/2017 | Asia / China | EPI1193683 \| A/Shanghai/11/2017 | EPI_ISL_302536 |
| A/Shanghai/10/2017 | Asia / China | EPI1193675 \| A/Shanghai/10/2017 | EPI_ISL_302535 |
| A/Shanghai/1/2016 | Asia / China | EPI1193667 \| A/Shanghai/1/2016 | EPI_ISL_302534 |
| A/Shanghai/28/2017 | Asia / China | EPI1193660 \| A/Shanghai/28/2017 | EPI_ISL_302533 |
| A/Shanghai/59/2017 | Asia / China | EPI1193656 \| A/Shanghai/59/2017 | EPI_ISL_302532 |
| A/Shanghai/60/2017 | Asia / China | EPI1193652 \| A/Shanghai/60/2017 | EPI_ISL_302531 |
| A/Shanghai/58/2017 | Asia / China | EPI1193648 \| A/Shanghai/58/2017 | EPI_ISL_302530 |
| A/Shanghai/23/2017 | Asia / China | EPI1193643 \| A/Shanghai/23/2017 | EPI_ISL_302529 |
| A/Shanghai/54/2017 | Asia / China | EPI1193636 \| A/Shanghai/54/2017 | EPI_ISL_302528 |
| A/Shanghai/27/2017 | Asia / China |  | EPI_ISL_302527 |
| A/Shanxi-Yingze/1692/2017 | Asia / China / Shanxi | EPI1168479 \| 2018-CX0170.4 | EPI_ISL_296984 |
| A/Neimenggu-Wulanhaote/1765/2017 | Asia / China / Neimenggu | EPI1168473 \| 2018-CX0172.4 | EPI_ISL_296982 |
| A/Shaanxi-Wangyi/1974/2017 | Asia / China / Shaanxi | EPI1168470 \| 2018-CX0173.4 | EPI_ISL_296981 |
| A/Liaoning-Donggang/1641/2017 | Asia / China / Liaoning | EPI1168467 \| 2018-CX0174.4 | EPI_ISL_296980 |
| A/Hebei-Hejian/1820/2017 | Asia / China / Hebei | EPI1168464 \| 2018-CX0175.4 | EPI_ISL_296979 |
| A/Guizhou-Xinyi/11038/2017 | Asia / China / Guizhou | EPI1168461 \| 2018-CX0176.4 | EPI_ISL_296978 |
| A/Xinjiang-Aletai/1605/2017 | Asia / China / Xinjiang | EPI1168455 \| 2018-CX0178.4 | EPI_ISL_296976 |
| A/Liaoning-Xinlongtai/1374/2017 | Asia / China / Liaoning | EPI1136335 \| 2017-CX2114.4 | EPI_ISL_290641 |
| A/Chongqing-Yuzhong/11766/2017 | Asia / China / Chongqing | EPI1136332 \| 2017-CX2115.4 | EPI_ISL_290640 |
| A/Anhui-Tianjiaan/1755/2017 | Asia / China / Anhui | EPI1136329 \| 2017-CX2116.4 | EPI_ISL_290639 |
| A/Sichuan-Jinjiang/2205/2017 | Asia / China / Sichuan | EPI1136326 \| 2017-CX2117.4 | EPI_ISL_290638 |
| A/Beijing-Xicheng/12002/2017 | Asia / China / Beijing | EPI1136323 \| 2017-CX2118.4 | EPI_ISL_290637 |
| A/Henan-Ruyang/1415/2017 | Asia / China / Henan | EPI1136320 \| 2017-CX2119.4 | EPI_ISL_290636 |
| A/Hubei-Xiling/37/2017 | Asia / China / Hubei | EPI1136317 \| 2017-CX2120.4 | EPI_ISL_290635 |
| A/Hubei-Maojian/11412/2017 | Asia / China / Hubei | EPI1136314 \| 2017-CX2121.4 | EPI_ISL_290634 |
| A/Henan-Zhaoling/1467/2017 | Asia / China / Henan | EPI1136311 \| 2017-CX2122.4 | EPI_ISL_290633 |
| A/Qinghai-Chengdong/1768/2017 | Asia / China / Qinghai | EPI1136308 \| 2017-CX2123.4 | EPI_ISL_290632 |
| A/Qinghai-Chengdong/1805/2017 | Asia / China / Qinghai | EPI1136305 \| 2017-CX2124.4 | EPI_ISL_290631 |
| A/Guizhou-Nanming/12448/2017 | Asia / China / Guizhou | EPI1136302 \| 2017-CX2125.4 | EPI_ISL_290630 |
| A/Sichuan-Shunqing/1813/2017 | Asia / China / Sichuan | EPI1136299 \| 2017-CX2126.4 | EPI_ISL_290629 |
| A/Liaoning-Yinzhou/1399/2017 | Asia / China / Liaoning | EPI1136296 \| 2017-CX2127.4 | EPI_ISL_290628 |
| A/Zhejiang-Nanhu/1745/2017 | Asia / China / Zhejiang | EPI1136293 \| 2017-CX2128.4 | EPI_ISL_290627 |
| A/Fujian-Siming/1748/2017 | Asia / China / Fujian | EPI1136290 \| 2017-CX2129.4 | EPI_ISL_290626 |
| A/Fujian-Licheng/1729/2017 | Asia / China / Fujian | EPI1136287 \| 2017-CX2130.4 | EPI_ISL_290625 |
| A/Guizhou-Douyun/1790/2017 | Asia / China / Guizhou | EPI1136284 \| 2017-CX2131.4 | EPI_ISL_290624 |
| A/Beijing-Huairou/11571/2017 | Asia / China / Beijing | EPI1136281 \| 2017-CX2132.4 | EPI_ISL_290623 |
| A/Shanghai-Fengxian/1811/2017 | Asia / China / Shanghai | EPI1136278 \| 2017-CX2133.4 | EPI_ISL_290622 |
| A/Hunan-Hecheng/1826/2017 | Asia / China / Hunan | EPI1136275 \| 2017-CX2134.4 | EPI_ISL_290621 |
| A/Beijing-Chaoyang/12560/2017 | Asia / China / Beijing | EPI1136272 \| 2017-CX2136.4 | EPI_ISL_290620 |
| A/Guizhou-Xixiu/1777/2017 | Asia / China / Guizhou | EPI1136269 \| 2017-CX2137.4 | EPI_ISL_290619 |
| A/Shanghai-Pudongxin/11764/2017 | Asia / China / Shanghai | EPI1136266 \| 2017-CX2138.4 | EPI_ISL_290618 |
| A/Liaoning-Heping/1411/2017 | Asia / China / Liaoning | EPI1136263 \| 2017-CX2139.4 | EPI_ISL_290617 |
| A/Hunan-Heshan/1700/2017 | Asia / China / Hunan | EPI1136260 \| 2017-CX2140.4 | EPI_ISL_290616 |
| A/Jiangxi-Xinzhou/1803/2017 | Asia / China / Jiangxi | EPI1136257 \| 2017-CX2141.4 | EPI_ISL_290615 |
| A/Yunnan-Hongta/11225/2017 | Asia / China / Yunnan | EPI1136254 \| 2017-CX2142.4 | EPI_ISL_290614 |
| A/Shanghai-Huangpu/11967/2017 | Asia / China / Shanghai | EPI1136209 \| 2017-CX2169.4 | EPI_ISL_290599 |
| A/Jiangsu-Sucheng/1834/2017 | Asia / China / Jiangsu | EPI1136206 \| 2017-CX2170.4 | EPI_ISL_290598 |
| A/Sichuan-Qingyang/11804/2017 | Asia / China / Sichuan | EPI1136203 \| 2017-CX2171.4 | EPI_ISL_290597 |
| A/Shanghai-Chongming/1906/2017 | Asia / China / Shanghai | EPI1136200 \| 2017-CX2172.4 | EPI_ISL_290596 |
| A/Beijing-Chaoyang/12911/2017 | Asia / China / Beijing | EPI1136197 \| 2017-CX2173.4 | EPI_ISL_290595 |
| A/Hubei-Xianan/1702/2017 | Asia / China / Hubei | EPI1125253 \| 2017-CX2018.4 | EPI_ISL_288681 |
| A/Guizhou-Huichuan/1636/2017 | Asia / China / Guizhou | EPI1125250 \| 2017-CX2019.4 | EPI_ISL_288680 |
| A/Hubei-Maojian/11402/2017 | Asia / China / Hubei | EPI1125247 \| 2017-CX2022.4 | EPI_ISL_288679 |
| A/Shanghai-Songjiang/1635/2017 | Asia / China / Shanghai | EPI1125241 \| 2017-CX2062.4 | EPI_ISL_288677 |
| A/Yunnan-Mengzi/1724/2017 | Asia / China / Yunnan | EPI1106250 \| 2017-CX2015.4 | EPI_ISL_285903 |
| A/Hubei-Xiaonan/1779/2017 | Asia / China / Hubei | EPI1106244 \| 2017-CX2017.4 | EPI_ISL_285901 |
| A/Fujian-Sanyuan/2691/2017 | Asia / China / Fujian | EPI1106241 \| 2017-CX2020.4 | EPI_ISL_285900 |
| A/Zhejiang-Nanhu/1679/2017 | Asia / China / Zhejiang | EPI1106238 \| 2017-CX2021.4 | EPI_ISL_285899 |
| A/Shandong-Zhifu/1128/2017 | Asia / China | EPI1067714 \| A/Shandong-Zhifu/1128/2017 | EPI_ISL_279451 |
| A/Shandong-Fushan/1157/2017 | Asia / China | EPI1067713 \| A/Shandong-Fushan/1157/2017 | EPI_ISL_279450 |
| A/Shandong-Zhifu/1125/2017 | Asia / China | EPI1067712 \| A/Shandong-Zhifu/1125/2017 | EPI_ISL_279449 |
| A/Shandong-Zhifu/1159/2017 | Asia / China | EPI1067711 \| A/Shandong-Zhifu/1159/2017 | EPI_ISL_279448 |
| A/Shandong-Zhifu/1175/2017 | Asia / China | EPI1067710 \| A/Shandong-Zhifu/1175/2017 | EPI_ISL_279447 |
| A/Shandong-Fushan/1183/2017 | Asia / China | EPI1067709 \| A/Shandong-Fushan/1183/2017 | EPI_ISL_279446 |
| A/Shandong-Zhifu/1174/2017 | Asia / China | EPI1067708 \| A/Shandong-Zhifu/1174/2017 | EPI_ISL_279445 |
| A/Shandong-Zhifu/1158/2017 | Asia / China | EPI1067707 \| A/Shandong-Zhifu/1158/2017 | EPI_ISL_279444 |
| A/Shandong-Zhifu/148/2017 | Asia / China | EPI1067706 \| A/Shandong-Zhifu/148/2017 | EPI_ISL_279443 |
| A/Shandong-Fushan/164/2017 | Asia / China | EPI1067705 \| A/Shandong-Fushan/164/2017 | EPI_ISL_279442 |
| A/Shandong-Zhifu/154/2017 | Asia / China | EPI1067704 \| A/Shandong-Zhifu/154/2017 | EPI_ISL_279441 |
| A/Shandong-Zhifu/1666/2016 | Asia / China | EPI1067703 \| A/Shandong-Zhifu/1666/2016 | EPI_ISL_279440 |
| A/Shandong-Fushan/1726/2016 | Asia / China | EPI1067702 \| A/Shandong-Fushan/1726/2016 | EPI_ISL_279439 |
| A/Shandong-Fushan/1721/2016 | Asia / China | EPI1067701 \| A/Shandong-Fushan/1721/2016 | EPI_ISL_279438 |
| A/Shandong-Fushan/1711/2016 | Asia / China | EPI1067700 \| A/Shandong-Fushan/1711/2016 | EPI_ISL_279437 |
| A/Shandong-Fushan/1631/2016 | Asia / China | EPI1067699 \| A/Shandong-Fushan/1631/2016 | EPI_ISL_279436 |
| A/Shandong-Zhifu/164/2017 | Asia / China | EPI1067698 \| A/Shandong-Zhifu/164/2017 | EPI_ISL_279435 |
| A/Shandong-Zhifu/149/2017 | Asia / China | EPI1067697 \| A/Shandong-Zhifu/149/2017 | EPI_ISL_279434 |
| A/Shandong-Zhifu/162/2017 | Asia / China | EPI1067696 \| A/Shandong-Zhifu/162/2017 | EPI_ISL_279433 |
| A/Shandong-Zhifu/150/2017 | Asia / China | EPI1067695 \| A/Shandong-Zhifu/150/2017 | EPI_ISL_279432 |
| A/Shandong-Fushan/148/2017 | Asia / China | EPI1067694 \| A/Shandong-Fushan/148/2017 | EPI_ISL_279431 |
| A/Shandong-Fushan/1257/2016 | Asia / China | EPI1067693 \| A/Shandong-Fushan/1257/2016 | EPI_ISL_279430 |
| A/Shandong-Zhifu/1185/2016 | Asia / China | EPI1067692 \| A/Shandong-Zhifu/1185/2016 | EPI_ISL_279429 |
| A/Shandong-Fushan/1182/2016 | Asia / China | EPI1067691 \| A/Shandong-Fushan/1182/2016 | EPI_ISL_279428 |
| A/Shandong-Zhifu/164/2016 | Asia / China | EPI1067690 \| A/Shandong-Zhifu/164/2016 | EPI_ISL_279427 |
| A/Jiangxi-Jizhou/1488/2017 | Asia / China / Jiangxi | EPI1062976 \| 2017-CX1479.4 | EPI_ISL_278733 |
| A/Shanghai-Pudongxin/1987/2017 | Asia / China / Shanghai | EPI1062973 \| 2017-CX1480.4 | EPI_ISL_278732 |
| A/Guangdong-Yuancheng/1471/2017 | Asia / China / Guangdong | EPI1062970 \| 2017-CX1481.4 | EPI_ISL_278731 |
| A/Heilongjiang-Beilin/1304/2017 | Asia / China / Heilongjiang | EPI1062967 \| 2017-CX1482.4 | EPI_ISL_278730 |
| A/Guangxi-Jiangzhou/1518/2017 | Asia / China / Guangxi | EPI1062964 \| 2017-CX1483.4 | EPI_ISL_278729 |
| A/Jiangsu-Runzhou/1553/2017 | Asia / China / Jiangsu | EPI1062961 \| 2017-CX1484.4 | EPI_ISL_278728 |
| A/Zhejiang-Jiaojiang/1255/2017 | Asia / China / Zhejiang | EPI1062958 \| 2017-CX1486.4 | EPI_ISL_278727 |
| A/Jilin-Ningjiang/1298/2017 | Asia / China / Jilin | EPI1062955 \| 2017-CX1487.4 | EPI_ISL_278726 |
| A/Henan-Jiefang/1275/2017 | Asia / China / Henan | EPI1062952 \| 2017-CX1489.4 | EPI_ISL_278725 |
| A/Hunan-Louxin/5100/2017 | Asia / China / Hunan | EPI1062949 \| 2017-CX1490.4 | EPI_ISL_278724 |
| A/Jilin-Ningjiang/1299/2017 | Asia / China / Jilin | EPI1062946 \| 2017-CX1491.4 | EPI_ISL_278723 |
| A/Fujian-Licheng/1354/2017 | Asia / China / Fujian | EPI1062943 \| 2017-CX1492.4 | EPI_ISL_278722 |
| A/Yunnan-Linxiang/1507/2017 | Asia / China / Yunnan | EPI1062940 \| 2017-CX1494.4 | EPI_ISL_278721 |
| A/Hunan-Jishou/11056/2017 | Asia / China / Hunan | EPI1062937 \| 2017-CX1495.4 | EPI_ISL_278720 |
| A/Shanghai-Xuhui/1373/2017 | Asia / China / Shanghai | EPI1062934 \| 2017-CX1496.4 | EPI_ISL_278719 |
| A/Jiangxi-Donghu/1850/2017 | Asia / China / Jiangxi | EPI1062931 \| 2017-CX1497.4 | EPI_ISL_278718 |
| A/Yunnan-Xianggelila/1375/2017 | Asia / China / Yunnan | EPI1062928 \| 2017-CX1498.4 | EPI_ISL_278717 |
| A/Fujian-Chengxiang/32/2017 | Asia / China / Fujian | EPI1062925 \| 2017-CX1499.4 | EPI_ISL_278716 |
| A/Hunan-Yanfeng/1478/2017 | Asia / China / Hunan | EPI1062922 \| 2017-CX1500.4 | EPI_ISL_278715 |
| A/Guangdong-Yuncheng/1857/2017 | Asia / China / Guangdong | EPI1062919 \| 2017-CX1501.4 | EPI_ISL_278714 |
| A/Hunan-Louxin/562/2017 | Asia / China / Hunan | EPI1062916 \| 2017-CX1503.4 | EPI_ISL_278713 |
| A/Hunan-Louxin/1333/2017 | Asia / China / Hunan | EPI1062913 \| 2017-CX1504.4 | EPI_ISL_278712 |
| A/Anhui-Xiangshan/1381/2017 | Asia / China / Anhui | EPI1062910 \| 2017-CX1507.4 | EPI_ISL_278711 |
| A/Chongqing-Yuzhong/361/2017 | Asia / China / Chongqing | EPI1062907 \| 2017-CX1508.4 | EPI_ISL_278710 |
| A/Qinghai-Gangcha/1235/2017 | Asia / China / Qinghai | EPI1062904 \| 2017-CX1509.4 | EPI_ISL_278709 |
| A/Yunnan-Linxiang/1611/2017 | Asia / China / Yunnan | EPI1062901 \| 2017-CX1510.4 | EPI_ISL_278708 |
| A/Hunan-Jishou/11139/2017 | Asia / China / Hunan | EPI1062898 \| 2017-CX1511.4 | EPI_ISL_278707 |
| A/Fujian-Licheng/1448/2017 | Asia / China / Fujian | EPI1062895 \| 2017-CX1512.4 | EPI_ISL_278706 |
| A/Guangxi-Gangbei/1482/2017 | Asia / China / Guangxi | EPI1062892 \| 2017-CX1513.4 | EPI_ISL_278705 |
| A/Macao/615046/2016 | Asia / China / Macao | EPI1062889 \| 2017-CX1514.4 | EPI_ISL_278704 |
| A/Jiangsu-Jurong/1541/2017 | Asia / China / Jiangsu | EPI1062808 \| 2017-CX1761.4 | EPI_ISL_278677 |
| A/Jiangsu-Yandou/16/2017 | Asia / China / Jiangsu | EPI1062805 \| 2017-CX1791.4 | EPI_ISL_278676 |
| A/Guangdong-Wujiang/363/2017 | Asia / China / Guangdong | EPI1062730 \| 2017-CX1793.4 | EPI_ISL_278651 |
| A/Fujian-Fengze/1491/2017 | Asia / China / Fujian | EPI1062727 \| 2017-CX1794.4 | EPI_ISL_278650 |
| A/Zhejiang-Haishu/11108/2017 | Asia / China / Zhejiang | EPI1062724 \| 2017-CX1795.4 | EPI_ISL_278649 |
| A/Zhejiang-Haishu/11068/2017 | Asia / China / Zhejiang | EPI1062721 \| 2017-CX1796.4 | EPI_ISL_278648 |
| A/Yunnan-Hongta/1723/2017 | Asia / China / Yunnan | EPI1062718 \| 2017-CX1797.4 | EPI_ISL_278647 |
| A/Yunnan-Jinghong/1653/2017 | Asia / China / Yunnan | EPI1062715 \| 2017-CX1798.4 | EPI_ISL_278646 |
| A/Shanghai-Fengxian/1599/2017 | Asia / China / Shanghai | EPI1062712 \| 2017-CX1799.4 | EPI_ISL_278645 |
| A/Sichuan-Gaoxin/1562/2017 | Asia / China / Sichuan | EPI1062709 \| 2017-CX1800.4 | EPI_ISL_278644 |
| A/Hubei-Songzi/1568/2017 | Asia / China / Hubei | EPI1062706 \| 2017-CX1801.4 | EPI_ISL_278643 |
| A/Hubei-Shashi/1599/2017 | Asia / China / Hubei | EPI1062703 \| 2017-CX1802.4 | EPI_ISL_278642 |
| A/Hubei-Fancheng/1278/2017 | Asia / China / Hubei | EPI1062700 \| 2017-CX1803.4 | EPI_ISL_278641 |
| A/Hubei-Xiangcheng/1610/2017 | Asia / China / Hubei | EPI1062697 \| 2017-CX1804.4 | EPI_ISL_278640 |
| A/Hubei-Wujiagang/1567/2017 | Asia / China / Hubei | EPI1062694 \| 2017-CX1805.4 | EPI_ISL_278639 |
| A/Hubei-Xiling/1196/2017 | Asia / China / Hubei | EPI1062691 \| 2017-CX1806.4 | EPI_ISL_278638 |
| A/Zhejiang-Kecheng/1574/2017 | Asia / China / Zhejiang | EPI1062688 \| 2017-CX1807.4 | EPI_ISL_278637 |
| A/Zhejiang-Haishu/11168/2017 | Asia / China / Zhejiang | EPI1062685 \| 2017-CX1808.4 | EPI_ISL_278636 |
| A/Guangdong-Pengjiang/1707/2017 | Asia / China / Guangdong | EPI1062682 \| 2017-CX1809.4 | EPI_ISL_278635 |
| A/Guangdong-Huicheng/1557/2017 | Asia / China / Guangdong | EPI1062679 \| 2017-CX1810.4 | EPI_ISL_278634 |
| A/Guangdong-Haizhu/1581/2017 | Asia / China / Guangdong | EPI1062676 \| 2017-CX1811.4 | EPI_ISL_278633 |
| A/Guangdong-Chancheng/1575/2017 | Asia / China / Guangdong | EPI1062673 \| 2017-CX1812.4 | EPI_ISL_278632 |
| A/Guangdong-Dongwanbendi/1596/2017 | Asia / China / Guangdong | EPI1062670 \| 2017-CX1813.4 | EPI_ISL_278631 |
| A/Guangxi-Yuzhou/1622/2017 | Asia / China / Guangxi | EPI1062667 \| 2017-CX1814.4 | EPI_ISL_278630 |
| A/Guangxi-Rongshuimiaozuzizhi/315/2017 | Asia / China / Guangxi | EPI1062664 \| 2017-CX1815.4 | EPI_ISL_278629 |
| A/Guangxi-Chengzhong/1672/2017 | Asia / China / Guangxi | EPI1062661 \| 2017-CX1816.4 | EPI_ISL_278628 |
| A/Guangxi-Youjiang/1592/2017 | Asia / China / Guangxi | EPI1062658 \| 2017-CX1817.4 | EPI_ISL_278627 |
| A/Guangxi-Gangbei/1582/2017 | Asia / China / Guangxi | EPI1062655 \| 2017-CX1818.4 | EPI_ISL_278626 |
| A/Guangxi-Babu/1606/2017 | Asia / China / Guangxi | EPI1062652 \| 2017-CX1819.4 | EPI_ISL_278625 |
| A/Hunan-Wuling/11222/2017 | Asia / China / Hunan | EPI1062649 \| 2017-CX1820.4 | EPI_ISL_278624 |
| A/Hunan-Jishou/11465/2017 | Asia / China / Hunan | EPI1062646 \| 2017-CX1821.4 | EPI_ISL_278623 |
| A/Hunan-Yanfeng/1655/2017 | Asia / China / Hunan | EPI1062643 \| 2017-CX1822.4 | EPI_ISL_278622 |
| A/Hunan-Zhuhui/1618/2017 | Asia / China / Hunan | EPI1062640 \| 2017-CX1823.4 | EPI_ISL_278621 |
| A/Shanghai-Jiading/1555/2017 | Asia / China / Shanghai | EPI1062637 \| 2017-CX1824.4 | EPI_ISL_278620 |
| A/Shanghai-Fengxian/1618/2017 | Asia / China / Shanghai | EPI1062634 \| 2017-CX1825.4 | EPI_ISL_278619 |
| A/Yunnan-Hongta/1728/2017 | Asia / China / Yunnan | EPI1062631 \| 2017-CX1826.4 | EPI_ISL_278618 |
| A/Yunnan-Jinghong/1673/2017 | Asia / China / Yunnan | EPI1062628 \| 2017-CX1827.4 | EPI_ISL_278617 |
| A/Yunnan-Linxiang/1816/2017 | Asia / China / Yunnan | EPI1062625 \| 2017-CX1828.4 | EPI_ISL_278616 |
| A/Fujian-Tongan/1533/2017 | Asia / China / Fujian | EPI1062622 \| 2017-CX1829.4 | EPI_ISL_278615 |
| A/Fujian-Pinghe/313/2017 | Asia / China / Fujian | EPI1062619 \| 2017-CX1830.4 | EPI_ISL_278614 |
| A/Fujian-Xiangcheng/1625/2017 | Asia / China / Fujian | EPI1062616 \| 2017-CX1831.4 | EPI_ISL_278613 |
| A/Jiangxi-Zhushan/1562/2017 | Asia / China / Jiangxi | EPI1062613 \| 2017-CX1832.4 | EPI_ISL_278612 |
| A/Jiangxi-Xihu/1619/2017 | Asia / China / Jiangxi | EPI1062610 \| 2017-CX1833.4 | EPI_ISL_278611 |
| A/Hunan-Hecheng/1648/2017 | Asia / China / Hunan | EPI1062607 \| 2017-CX1835.4 | EPI_ISL_278610 |
| A/Hunan-Kaifu/1574/2017 | Asia / China / Hunan | EPI1062604 \| 2017-CX1836.4 | EPI_ISL_278609 |
| A/Jiangxi-Zhanggong/1549/2017 | Asia / China / Jiangxi | EPI1062601 \| 2017-CX1837.4 | EPI_ISL_278608 |
| A/Jiangxi-Yushui/1571/2017 | Asia / China / Jiangxi | EPI1062598 \| 2017-CX1838.4 | EPI_ISL_278607 |
| A/Jiangxi-Zhanggong/1513/2017 | Asia / China / Jiangxi | EPI1062595 \| 2017-CX1839.4 | EPI_ISL_278606 |
| A/Guangdong-Jiangcheng/1563/2017 | Asia / China / Guangdong | EPI1062592 \| 2017-CX1840.4 | EPI_ISL_278605 |
| A/Hubei-Jiangan/1575/2017 | Asia / China / Hubei | EPI1062589 \| 2017-CX1841.4 | EPI_ISL_278604 |
| A/Guangdong-Chengqu/1649/2017 | Asia / China / Guangdong | EPI1062586 \| 2017-CX1843.4 | EPI_ISL_278603 |
| A/Guangdong-Yuancheng/1587/2017 | Asia / China / Guangdong | EPI1062583 \| 2017-CX1844.4 | EPI_ISL_278602 |
| A/Guangdong-Lechang/33/2017 | Asia / China / Guangdong | EPI1062580 \| 2017-CX1845.4 | EPI_ISL_278601 |
| A/Guangdong-Yingde/37/2017 | Asia / China / Guangdong | EPI1062577 \| 2017-CX1846.4 | EPI_ISL_278600 |
| A/Guangdong-Qingcheng/1608/2017 | Asia / China / Guangdong | EPI1062574 \| 2017-CX1847.4 | EPI_ISL_278599 |
| A/Sichuan-Jiangyang/11227/2017 | Asia / China / Sichuan | EPI1062571 \| 2017-CX1848.4 | EPI_ISL_278598 |
| A/Sichuan-Gaoxin/1586/2017 | Asia / China / Sichuan | EPI1062568 \| 2017-CX1849.4 | EPI_ISL_278597 |
| A/Hubei-Jiangan/1578/2017 | Asia / China / Hubei | EPI1062565 \| 2017-CX1850.4 | EPI_ISL_278596 |
| A/Shanghai-Pudongxin/11115/2017 | Asia / China / Shanghai | EPI1062562 \| 2017-CX1851.4 | EPI_ISL_278595 |
| A/Guangdong-Duanzhou/1607/2017 | Asia / China / Guangdong | EPI1062559 \| 2017-CX1852.4 | EPI_ISL_278594 |
| A/Guangdong-Longgang/385/2017 | Asia / China / Guangdong | EPI1062556 \| 2017-CX1853.4 | EPI_ISL_278593 |
| A/Guangdong-Wujiang/321/2017 | Asia / China / Guangdong | EPI1062553 \| 2017-CX1854.4 | EPI_ISL_278592 |
| A/Sichuan-Shizhong/1653/2017 | Asia / China / Sichuan | EPI1062550 \| 2017-CX1855.4 | EPI_ISL_278591 |
| A/Sichuan-Chuanshan/11005/2017 | Asia / China / Sichuan | EPI1062547 \| 2017-CX1856.4 | EPI_ISL_278590 |
| A/Guangxi-Wanxiu/1486/2017 | Asia / China / Guangxi | EPI1062544 \| 2017-CX1857.4 | EPI_ISL_278589 |
| A/Guangxi-Yuzhou/1527/2017 | Asia / China / Guangxi | EPI1062541 \| 2017-CX1858.4 | EPI_ISL_278588 |
| A/Guangxi-Longan/1539/2017 | Asia / China / Guangxi | EPI1062538 \| 2017-CX1859.4 | EPI_ISL_278587 |
| A/Guangdong-Yuncheng/1964/2017 | Asia / China / Guangdong | EPI1062535 \| 2017-CX1861.4 | EPI_ISL_278586 |
| A/Chongqing-Yuzhong/11141/2017 | Asia / China / Chongqing | EPI1062532 \| 2017-CX1862.4 | EPI_ISL_278585 |
| A/Hunan-Louxin/1546/2017 | Asia / China / Hunan | EPI1062529 \| 2017-CX1863.4 | EPI_ISL_278584 |
| A/Hunan-Yuhua/1938/2017 | Asia / China / Hunan | EPI1062526 \| 2017-CX1864.4 | EPI_ISL_278583 |
| A/Hunan-Zhuhui/1502/2017 | Asia / China / Hunan | EPI1062523 \| 2017-CX1866.4 | EPI_ISL_278582 |
| A/Hunan-Yuhua/11098/2017 | Asia / China / Hunan | EPI1062520 \| 2017-CX1867.4 | EPI_ISL_278581 |
| A/Hunan-Jishou/11306/2017 | Asia / China / Hunan | EPI1062517 \| 2017-CX1868.4 | EPI_ISL_278580 |
| A/Guangdong-Chengqu/1585/2017 | Asia / China / Guangdong | EPI1062514 \| 2017-CX1869.4 | EPI_ISL_278579 |
| A/Guangdong-Maonan/1554/2017 | Asia / China / Guangdong | EPI1062511 \| 2017-CX1870.4 | EPI_ISL_278578 |
| A/Guangdong-Chancheng/1510/2017 | Asia / China / Guangdong | EPI1062508 \| 2017-CX1871.4 | EPI_ISL_278577 |
| A/Guangdong-Duanzhou/1555/2017 | Asia / China / Guangdong | EPI1062505 \| 2017-CX1874.4 | EPI_ISL_278576 |
| A/Sichuan-Qingyang/11132/2017 | Asia / China / Sichuan | EPI1062502 \| 2017-CX1875.4 | EPI_ISL_278575 |
| A/Jiangxi-Donghu/11087/2017 | Asia / China / Jiangxi | EPI1062499 \| 2017-CX1876.4 | EPI_ISL_278574 |
| A/Jiangxi-Donghu/11024/2017 | Asia / China / Jiangxi | EPI1062496 \| 2017-CX1877.4 | EPI_ISL_278573 |
| A/Jiangxi-Zhushan/1504/2017 | Asia / China / Jiangxi | EPI1062493 \| 2017-CX1878.4 | EPI_ISL_278572 |
| A/Fujian-Fengze/1507/2017 | Asia / China / Fujian | EPI1062490 \| 2017-CX1879.4 | EPI_ISL_278571 |
| A/Jiangsu-Sucheng/1513/2017 | Asia / China / Jiangsu | EPI1062487 \| 2017-CX1880.4 | EPI_ISL_278570 |
| A/Shanghai-Minxing/1482/2017 | Asia / China / Shanghai | EPI1062484 \| 2017-CX1882.4 | EPI_ISL_278569 |
| A/Shanghai-Jinshan/1514/2017 | Asia / China / Shanghai | EPI1062481 \| 2017-CX1883.4 | EPI_ISL_278568 |
| A/Shanghai-Fengxian/1536/2017 | Asia / China / Shanghai | EPI1062478 \| 2017-CX1884.4 | EPI_ISL_278567 |
| A/Guangdong-Pengjiang/1573/2017 | Asia / China / Guangdong | EPI1062475 \| 2017-CX1885.4 | EPI_ISL_278566 |
| A/Guangdong-Xiangzhou/1944/2017 | Asia / China / Guangdong | EPI1062472 \| 2017-CX1886.4 | EPI_ISL_278565 |
| A/Guangdong-Chikan/1496/2017 | Asia / China / Guangdong | EPI1062469 \| 2017-CX1888.4 | EPI_ISL_278564 |
| A/Yunnan-Hongta/1769/2017 | Asia / China / Yunnan | EPI1062433 \| 2017-CX1917.4 | EPI_ISL_278552 |
| A/Jiangsu-Haizhou/1692/2017 | Asia / China / Jiangsu | EPI1062430 \| 2017-CX1918.4 | EPI_ISL_278551 |
| A/Sichuan-Jinjiang/59/2017 | Asia / China / Sichuan | EPI1062427 \| 2017-CX1919.4 | EPI_ISL_278550 |
| A/Guangdong-Maonan/1520/2017 | Asia / China / Guangdong | EPI1062424 \| 2017-CX1920.4 | EPI_ISL_278549 |
| A/Guangdong-Chancheng/1618/2017 | Asia / China / Guangdong | EPI1062421 \| 2017-CX1921.4 | EPI_ISL_278548 |
| A/Beijing-Huairou/11323/2017 | Asia / China / Beijing | EPI1062418 \| 2017-CX1922.4 | EPI_ISL_278547 |
| A/Hunan-Yuhua/11237/2017 | Asia / China / Hunan | EPI1062415 \| 2017-CX1923.4 | EPI_ISL_278546 |
| A/Hubei-Dongbao/1631/2017 | Asia / China / Hubei | EPI1062412 \| 2017-CX1924.4 | EPI_ISL_278545 |
| A/Jiangxi-Zhushan/1611/2017 | Asia / China / Jiangxi | EPI1062409 \| 2017-CX1925.4 | EPI_ISL_278544 |
| A/Shanghai-Putuo/1623/2017 | Asia / China / Shanghai | EPI1062406 \| 2017-CX1926.4 | EPI_ISL_278543 |
| A/Hubei-Fancheng/1278/2017 | Asia / China / Hubei | EPI1062403 \| 2017-CX1927.4 | EPI_ISL_278542 |
| A/Guangdong-Pengjiang/1707/2017 | Asia / China / Guangdong | EPI1062400 \| 2017-CX1928.4 | EPI_ISL_278541 |
| A/Sichuan-Chuanshan/11005/2017 | Asia / China / Sichuan | EPI1062397 \| 2017-CX1929.4 | EPI_ISL_278540 |
| A/Hunan-Xinhua/523/2017 | Asia / China / Hunan | EPI1062394 \| 2017-CX1930.4 | EPI_ISL_278539 |
| A/Jiangsu-Sucheng/1489/2017 | Asia / China / Jiangsu | EPI1062391 \| 2017-CX1931.4 | EPI_ISL_278538 |
| A/Shanghai-Pudongxin/1987/2017 | Asia / China | EPI1058537 \| 3000484921_N8K8HQVO_v1_4 | EPI_ISL_277532 |
| A/Guangdong/264/2016 | Asia / China | EPI1034412 \| A/Guangdong/264/2016 | EPI_ISL_272716 |
| A/Guangdong/12/2016 | Asia / China | EPI1034411 \| A/Guangdong/12/2016 | EPI_ISL_272715 |
| A/Heilongjiang-Aihui/1152/2017 | Asia / China / Heilongjiang | EPI1030348 \| 2017-CX0847.4 | EPI_ISL_270748 |
| A/Henan-Shihe/1212/2017 | Asia / China / Henan | EPI1030345 \| 2017-CX0848.4 | EPI_ISL_270747 |
| A/Shaanxi-Weibin/5161/2017 | Asia / China / Shaanxi | EPI1030342 \| 2017-CX0849.4 | EPI_ISL_270746 |
| A/Guangdong-Maonan/1173/2017 | Asia / China / Guangdong | EPI1030339 \| 2017-CX0850.4 | EPI_ISL_270745 |
| A/Guizhou-Zhongshan/124/2017 | Asia / China / Guizhou | EPI1030336 \| 2017-CX0851.4 | EPI_ISL_270744 |
| A/Shandong-Laicheng/1763/2016 | Asia / China / Shandong | EPI1030333 \| 2017-CX0853.4 | EPI_ISL_270743 |
| A/Gansu-Chengguan/145/2017 | Asia / China / Gansu | EPI1030330 \| 2017-CX0854.4 | EPI_ISL_270742 |
| A/Guangxi-Gangbei/1207/2017 | Asia / China / Guangxi | EPI1030276 \| 2017-CX0989.4 | EPI_ISL_270724 |
| A/Liaoning-Mingshan/1188/2017 | Asia / China / Liaoning | EPI1030273 \| 2017-CX0991.4 | EPI_ISL_270723 |
| A/Tianjin-Baodi/1165/2017 | Asia / China | EPI1030270 \| 2017-CX0993.4 | EPI_ISL_270722 |
| A/Hebei-Yunhe/1101/2017 | Asia / China / Hebei | EPI1030198 \| 2017-CX0846.4 | EPI_ISL_270698 |
| A/Anhui-Xuanzhou/1179/2017 | Asia / China / Anhui | EPI1030195 \| 2017-CX0990.4 | EPI_ISL_270697 |
| A/Jiangsu-Yandou/16/2017 | Asia / China / Jiangsu | EPI1030009 \| 2017-CX1248.4 | EPI_ISL_270635 |
| A/Fujian-Licheng/1327/2017 | Asia / China / Fujian | EPI1030006 \| 2017-CX1249.4 | EPI_ISL_270634 |
| A/Shanghai-Huangpu/1730/2017 | Asia / China | EPI1030003 \| 2017-CX1250.4 | EPI_ISL_270633 |
| A/Guangdong-Duanzhou/1359/2017 | Asia / China / Guangdong | EPI1030000 \| 2017-CX1251.4 | EPI_ISL_270632 |
| A/Hunan-Jishou/1912/2017 | Asia / China / Hunan | EPI1029997 \| 2017-CX1252.4 | EPI_ISL_270631 |
| A/Anhui-Yaohai/1307/2017 | Asia / China / Anhui | EPI1029994 \| 2017-CX1253.4 | EPI_ISL_270630 |
| A/Yunnan-Chuxiong/1376/2017 | Asia / China / Yunnan | EPI1029991 \| 2017-CX1254.4 | EPI_ISL_270629 |
| A/Heilongjiang-Yichun/1567/2017 | Asia / China / Heilongjiang | EPI1029988 \| 2017-CX1255.4 | EPI_ISL_270628 |
| A/Guangdong-Duanzhou/1383/2017 | Asia / China / Guangdong | EPI1029985 \| 2017-CX1256.4 | EPI_ISL_270627 |
| A/Guangdong-Zhongshan/1411/2017 | Asia / China / Guangdong | EPI1029982 \| 2017-CX1257.4 | EPI_ISL_270626 |
| A/Shanghai-Huangpu/1818/2017 | Asia / China | EPI1029979 \| 2017-CX1258.4 | EPI_ISL_270625 |
| A/Fujian-Fengze/1353/2017 | Asia / China / Fujian | EPI1029976 \| 2017-CX1259.4 | EPI_ISL_270624 |
| A/Guangdong-Zhenjiang/1326/2017 | Asia / China / Guangdong | EPI1029916 \| 2017-CX1381.4 | EPI_ISL_270604 |
| A/Ningxia-Yuanzhou/1657/2016 | Asia / China | EPI976575 \| 3000684654_000J36CF_v1_4 | EPI_ISL_259165 |
| A/Zhejiang-Nanhu/1975/2016 | Asia / China | EPI955883 \| 3000684653_N8K8YC0B_v1_4 | EPI_ISL_255349 |
| A/Guizhou-Qingzhen/1968/2016 | Asia / China | EPI955719 \| 3000684652_N8K8YC06_v1_4 | EPI_ISL_255328 |
| A/Anhui-Yingjiang/12306/2016 | Asia / China / Anhui | EPI944007 \| 2017-CX0340.4 | EPI_ISL_253303 |
| A/Beijing-Xicheng/1136/2017 | Asia / China / Beijing | EPI943968 \| 2017-CX0429.4 | EPI_ISL_253290 |
| A/Shaanxi-Linwei/163/2017 | Asia / China / Shaanxi | EPI943965 \| 2017-CX0430.4 | EPI_ISL_253289 |
| A/Heilongjiang-Aihui/15/2017 | Asia / China / Heilongjiang | EPI943962 \| 2017-CX0431.4 | EPI_ISL_253288 |
| A/Shandong-Laicheng/1760/2016 | Asia / China / Shandong | EPI943959 \| 2017-CX0432.4 | EPI_ISL_253287 |
| A/Shaanxi-Hanbin/11305/2016 | Asia / China / Shaanxi | EPI943956 \| 2017-CX0433.4 | EPI_ISL_253286 |
| A/Shaanxi-Hanbin/11305/2016 | Asia / China / Shaanxi | EPI943953 \| 2017-CX0435.4 | EPI_ISL_253285 |
| A/Xiamen/s228/2016 | Asia / China | EPI899648 \| A/Xiamen/s228/2016 | EPI_ISL_245489 |
| A/Xiamen/s175/2016 | Asia / China | EPI899647 \| A/Xiamen/s175/2016 | EPI_ISL_245488 |
| A/Xiamen/s200/2016 | Asia / China | EPI899646 \| A/Xiamen/s200/2016 | EPI_ISL_245487 |
| A/Xinjiang-Tianshan/1287/2016 | Asia / China / Xinjiang | EPI885873 \| 2016-CX2553.4 | EPI_ISL_242574 |
| A/Hubei-Jiangan/1719/2016 | Asia / China / Hubei | EPI885867 \| 2016-CX2870.4 | EPI_ISL_242572 |
| A/Hubei-Hongshan/1320/2016 | Asia / China / Hubei | EPI885864 \| 2016-CX2871.4 | EPI_ISL_242571 |
| A/Beijing-Huairou/12355/2016 | Asia / China / Beijing | EPI885861 \| 2016-CX3018.4 | EPI_ISL_242570 |
| A/Guizhou-Honghuagang/1840/2016 | Asia / China / Guizhou | EPI885858 \| 2016-CX3019.4 | EPI_ISL_242569 |
| A/Yunnan-Wenshan/1902/2016 | Asia / China / Yunnan | EPI885855 \| 2016-CX3020.4 | EPI_ISL_242568 |
| A/Tianjin-Beichen/1498/2016 | Asia / China / Tianjin | EPI885852 \| 2016-CX3021.4 | EPI_ISL_242567 |
| A/Sichuan-Wuhou/2207/2016 | Asia / China / Sichuan | EPI885849 \| 2016-CX3022.4 | EPI_ISL_242566 |
| A/Jiangxi-Zhushan/1869/2016 | Asia / China / Jiangxi | EPI885846 \| 2016-CX3023.4 | EPI_ISL_242565 |
| A/Beijing-Daxin/315/2016 | Asia / China / Beijing | EPI885843 \| 2016-CX3024.4 | EPI_ISL_242564 |
| A/Tianjin-Nankai/1973/2016 | Asia / China / Tianjin | EPI885840 \| 2016-CX3025.4 | EPI_ISL_242563 |
| A/Zhejiang-Yongkang/1879/2016 | Asia / China / Zhejiang | EPI885837 \| 2016-CX3026.4 | EPI_ISL_242562 |
| A/Chongqing-Shapingba/310/2016 | Asia / China / Chongqing | EPI885834 \| 2016-CX3028.4 | EPI_ISL_242561 |
| A/Zhejiang-Yongkang/1779/2016 | Asia / China / Zhejiang | EPI885831 \| 2016-CX3029.4 | EPI_ISL_242560 |
| A/Guangdong-Zhongshan/1956/2016 | Asia / China / Guangdong | EPI885828 \| 2016-CX3030.4 | EPI_ISL_242559 |
| A/Tianjin-Nankai/1774/2016 | Asia / China / Tianjin | EPI885825 \| 2016-CX3031.4 | EPI_ISL_242558 |
| A/Jiangxi-Zhushan/1680/2016 | Asia / China / Jiangxi | EPI885822 \| 2016-CX3032.4 | EPI_ISL_242557 |
| A/Yunnan-Wenshan/1859/2016 | Asia / China / Yunnan | EPI885819 \| 2016-CX3034.4 | EPI_ISL_242556 |
| A/Shanghai-Hongkou/1792/2016 | Asia / China / Shanghai | EPI885816 \| 2016-CX3035.4 | EPI_ISL_242555 |
| A/Beijing-Xicheng/12534/2016 | Asia / China / Beijing | EPI885813 \| 2016-CX3036.4 | EPI_ISL_242554 |
| A/Jiangxi-Donghu/11758/2016 | Asia / China / Jiangxi | EPI885810 \| 2016-CX3037.4 | EPI_ISL_242553 |
| A/Sichuan-Wuhou/2202/2016 | Asia / China / Sichuan | EPI885807 \| 2016-CX3038.4 | EPI_ISL_242552 |
| A/Hunan-Yueyanglou/1846/2016 | Asia / China / Hunan | EPI885804 \| 2016-CX3039.4 | EPI_ISL_242551 |
| A/Hunan-Suxian/1980/2016 | Asia / China / Hunan | EPI885801 \| 2016-CX3040.4 | EPI_ISL_242550 |
| A/Hubei-Dongbao/1758/2016 | Asia / China / Hubei | EPI885798 \| 2016-CX3041.4 | EPI_ISL_242549 |
| A/Fujian-Gulou/11611/2016 | Asia / China / Fujian | EPI885795 \| 2016-CX3042.4 | EPI_ISL_242548 |
| A/Guangdong-Dongwanbendi/5342/2016 | Asia / China / Guangdong | EPI885792 \| 2016-CX3043.4 | EPI_ISL_242547 |
| A/Zhejiang-Wuxin/1981/2016 | Asia / China / Zhejiang | EPI885693 \| 2017-CX0048.4 | EPI_ISL_242514 |
| A/Jiangsu-Nanchang/1968/2016 | Asia / China / Jiangsu | EPI885690 \| 2017-CX0049.4 | EPI_ISL_242513 |
| A/Hunan-Yuhu/1884/2016 | Asia / China / Hunan | EPI885687 \| 2017-CX0050.4 | EPI_ISL_242512 |
| A/Zhejiang-Shangcheng/1931/2016 | Asia / China / Zhejiang | EPI885684 \| 2017-CX0051.4 | EPI_ISL_242511 |
| A/Liaoning-Xigang/1807/2016 | Asia / China / Liaoning | EPI885681 \| 2017-CX0052.4 | EPI_ISL_242510 |
| A/Sichuan-Shizhong/1673/2016 | Asia / China / Sichuan | EPI885678 \| 2017-CX0053.4 | EPI_ISL_242509 |
| A/Hebei-Luquan/1578/2016 | Asia / China / Hebei | EPI885675 \| 2017-CX0054.4 | EPI_ISL_242508 |
| A/Anhui-Yaohai/1917/2016 | Asia / China / Anhui | EPI885672 \| 2017-CX0055.4 | EPI_ISL_242507 |
| A/Gansu-Chengguan/11062/2016 | Asia / China / Gansu | EPI885669 \| 2017-CX0056.4 | EPI_ISL_242506 |
| A/Hunan-Wuling/12110/2016 | Asia / China / Hunan | EPI885666 \| 2017-CX0057.4 | EPI_ISL_242505 |
| A/Hubei-Wuchang/1590/2016 | Asia / China / Hubei | EPI885663 \| 2017-CX0058.4 | EPI_ISL_242504 |
| A/Fujian-Licheng/1868/2016 | Asia / China / Fujian | EPI885660 \| 2017-CX0059.4 | EPI_ISL_242503 |
| A/Shandong-Zhangqiu/1491/2016 | Asia / China / Shandong | EPI885657 \| 2017-CX0060.4 | EPI_ISL_242502 |
| A/Beijing-Dongcheng/11734/2016 | Asia / China / Beijing | EPI885654 \| 2017-CX0061.4 | EPI_ISL_242501 |
| A/Hunan-Yuhua/11799/2016 | Asia / China / Hunan | EPI885651 \| 2017-CX0062.4 | EPI_ISL_242500 |
| A/Yunnan-Wenshan/1869/2016 | Asia / China / Yunnan | EPI885648 \| 2017-CX0063.4 | EPI_ISL_242499 |
| A/Zhejiang-Yuecheng/1909/2016 | Asia / China / Zhejiang | EPI885645 \| 2017-CX0064.4 | EPI_ISL_242498 |
| A/Chongqing-Banan/1423/2016 | Asia / China / Chongqing | EPI885642 \| 2017-CX0065.4 | EPI_ISL_242497 |
| A/Jiangxi-Yushui/1822/2016 | Asia / China / Jiangxi | EPI885639 \| 2017-CX0066.4 | EPI_ISL_242496 |
| A/Beijing-Chaoyang/12907/2016 | Asia / China / Beijing | EPI885636 \| 2017-CX0067.4 | EPI_ISL_242495 |
| A/Hebei-Lunan/11129/2016 | Asia / China / Hebei | EPI885633 \| 2017-CX0068.4 | EPI_ISL_242494 |
| A/Yunnan-Hongta/11419/2016 | Asia / China / Yunnan | EPI885630 \| 2017-CX0069.4 | EPI_ISL_242493 |
| A/Guizhou-Qingzhen/1968/2016 | Asia / China / Guizhou | EPI885627 \| 2017-CX0070.4 | EPI_ISL_242492 |
| A/Sichuan-Jiangyang/11668/2016 | Asia / China / Sichuan | EPI885624 \| 2017-CX0072.4 | EPI_ISL_242491 |
| A/Guangdong-Yuancheng/1970/2016 | Asia / China / Guangdong | EPI885621 \| 2017-CX0073.4 | EPI_ISL_242490 |
| A/Xinjiang-Hutubi/1559/2016 | Asia / China / Xinjiang | EPI885618 \| 2017-CX0074.4 | EPI_ISL_242489 |
| A/Tianjin-Hedong/8265/2016 | Asia / China / Tianjin | EPI885615 \| 2017-CX0075.4 | EPI_ISL_242488 |
| A/Zhejiang-Nanhu/1975/2016 | Asia / China / Zhejiang | EPI885612 \| 2017-CX0076.4 | EPI_ISL_242487 |
| A/Henan-Zhongyuan/1578/2016 | Asia / China / Henan | EPI885609 \| 2017-CX0077.4 | EPI_ISL_242486 |
| A/Ningxia-Yuanzhou/1657/2016 | Asia / China / Ningxia | EPI885606 \| 2017-CX0078.4 | EPI_ISL_242485 |
| A/Zhejiang-Yongkang/1968/2016 | Asia / China / Zhejiang | EPI885603 \| 2017-CX0079.4 | EPI_ISL_242484 |
| A/Hunan-Yuhu/1966/2016 | Asia / China / Hunan | EPI885600 \| 2017-CX0080.4 | EPI_ISL_242483 |
| A/Sichuan-Shunqing/1920/2016 | Asia / China / Sichuan | EPI885597 \| 2017-CX0081.4 | EPI_ISL_242482 |
| A/Beijing-Huairou/12538/2016 | Asia / China / Beijing | EPI885594 \| 2017-CX0082.4 | EPI_ISL_242481 |
| A/Henan-Zhaoling/1597/2016 | Asia / China / Henan | EPI885591 \| 2017-CX0083.4 | EPI_ISL_242480 |
| A/Shaanxi-Hanbin/11182/2016 | Asia / China / Shaanxi | EPI885588 \| 2017-CX0084.4 | EPI_ISL_242479 |
| A/Tianjin-Hedong/1536/2016 | Asia / China / Tianjin | EPI885585 \| 2017-CX0085.4 | EPI_ISL_242478 |
| A/Anhui-Longzihu/1995/2016 | Asia / China / Anhui | EPI885582 \| 2017-CX0086.4 | EPI_ISL_242477 |
| A/Shaanxi-Changan/1569/2016 | Asia / China / Shaanxi | EPI885579 \| 2017-CX0087.4 | EPI_ISL_242476 |
| A/Gansu-Chengguan/11177/2016 | Asia / China / Gansu | EPI885576 \| 2017-CX0088.4 | EPI_ISL_242475 |
| A/Yunnan-Simao/11014/2016 | Asia / China / Yunnan | EPI885573 \| 2017-CX0089.4 | EPI_ISL_242474 |
| A/Anhui-Xuanzhou/11155/2016 | Asia / China / Anhui | EPI885570 \| 2017-CX0090.4 | EPI_ISL_242473 |
| A/Guangdong-Zhongshan/11131/2016 | Asia / China / Guangdong | EPI885567 \| 2017-CX0091.4 | EPI_ISL_242472 |
| A/Chongqing-Yuzhong/11421/2016 | Asia / China / Chongqing | EPI885564 \| 2017-CX0092.4 | EPI_ISL_242471 |
| A/Guangxi-Jinchengjiang/1996/2016 | Asia / China / Guangxi | EPI885561 \| 2017-CX0093.4 | EPI_ISL_242470 |
| A/Hunan-Yuhua/11939/2016 | Asia / China / Hunan | EPI885558 \| 2017-CX0094.4 | EPI_ISL_242469 |
| A/Jiangsu-Sucheng/1885/2016 | Asia / China / Jiangsu | EPI885555 \| 2017-CX0095.4 | EPI_ISL_242468 |
| A/Hunan-Louxin/120/2017 | Asia / China / Hunan | EPI885438 \| 2017-CX0248.4 | EPI_ISL_242429 |
| A/Henan-Shihe/123/2017 | Asia / China / Henan | EPI885435 \| 2017-CX0249.4 | EPI_ISL_242428 |
| A/Anhui-Shushan/3236/2016 | Asia / China / Anhui | EPI885432 \| 2017-CX0250.4 | EPI_ISL_242427 |
| A/Hubei-Wuchang/1678/2016 | Asia / China / Hubei | EPI885429 \| 2017-CX0251.4 | EPI_ISL_242426 |
| A/Guangxi-Qixin/328/2016 | Asia / China / Guangxi | EPI885426 \| 2017-CX0252.4 | EPI_ISL_242425 |
| A/Anhui-Jinghu/611/2017 | Asia / China / Anhui | EPI885423 \| 2017-CX0253.4 | EPI_ISL_242424 |
| A/Jilin-Longshan/15/2017 | Asia / China / Jilin | EPI885420 \| 2017-CX0254.4 | EPI_ISL_242423 |
| A/Neimenggu-Kundoulun/15/2017 | Asia / China / Neimenggu | EPI885417 \| 2017-CX0255.4 | EPI_ISL_242422 |
| A/Shandong-Rencheng/112/2017 | Asia / China / Shandong | EPI885414 \| 2017-CX0256.4 | EPI_ISL_242421 |
| A/Anhui-Luyang/3155/2016 | Asia / China / Anhui | EPI885411 \| 2017-CX0257.4 | EPI_ISL_242420 |
| A/Guizhou-Nanming/12878/2016 | Asia / China / Guizhou | EPI885408 \| 2017-CX0258.4 | EPI_ISL_242419 |
| A/Jiangsu-Sucheng/1309/2016 | Asia / China | EPI860079 \| 16S2376 | EPI_ISL_237889 |
| A/Jiangsu-Sucheng/1309/2016 | Asia / China | EPI843979 \| 3000481387_000J30CD_v1_4 | EPI_ISL_235538 |
| A/Jiangsu-Sucheng/1309/2016 | Asia / China | EPI836546 \| 3000481387_N8K8HD8U_v1_4 | EPI_ISL_234048 |
| A/Liaoning-Xishi/1371/2016 | Asia / China / Liaoning | EPI829555 \| 2016-CX2554.4 | EPI_ISL_232953 |
| A/Beijing-Xicheng/1955/2016 | Asia / China / Beijing | EPI829549 \| 2016-CX2551.4 | EPI_ISL_232951 |
| A/Sichuan-Jinjiang/8156/2016 | Asia / China / Sichuan | EPI829546 \| 2016-CX2550.4 | EPI_ISL_232950 |
| A/Hubei-Wuchang/1297/2016 | Asia / China / Hubei | EPI829543 \| 2016-CX2549.4 | EPI_ISL_232949 |
| A/Tianjin-Baodi/1211/2016 | Asia / China / Tianjin | EPI829540 \| 2016-CX2548.4 | EPI_ISL_232948 |
| A/Jiangsu-Danyang/1516/2016 | Asia / China / Jiangsu | EPI829537 \| 2016-CX2547.4 | EPI_ISL_232947 |
| A/Chongqing-Xiushantujiazumiaozuzizhi/330/2016 | Asia / China / Chongqing | EPI829534 \| 2016-CX2546.4 | EPI_ISL_232946 |
| A/Hunan-Xiangtan/1577/2016 | Asia / China / Hunan | EPI829531 \| 2016-CX2545.4 | EPI_ISL_232945 |
| A/Shanghai-Pudongxin/11154/2016 | Asia / China / Shanghai | EPI829528 \| 2016-CX2544.4 | EPI_ISL_232944 |
| A/Yunnan-Mengzi/1646/2016 | Asia / China / Yunnan | EPI829525 \| 2016-CX2543.4 | EPI_ISL_232943 |
| A/Jiangxi-Zhushan/1560/2016 | Asia / China / Jiangxi | EPI829522 \| 2016-CX2542.4 | EPI_ISL_232942 |
| A/Guangdong-Futian/1582/2016 | Asia / China / Guangdong | EPI829519 \| 2016-CX2541.4 | EPI_ISL_232941 |
| A/Henan-Jinshui/1222/2016 | Asia / China / Henan | EPI829516 \| 2016-CX2540.4 | EPI_ISL_232940 |
| A/Beijing-Chaoyang/1551/2016 | Asia / China / Beijing | EPI829513 \| 2016-CX2539.4 | EPI_ISL_232939 |
| A/Shandong-Shibei/1548/2016 | Asia / China / Shandong | EPI829510 \| 2016-CX2538.4 | EPI_ISL_232938 |
| A/Yunnan-Linxiang/1718/2016 | Asia / China / Yunnan | EPI817115 \| 2016-CX1845.4 | EPI_ISL_230739 |
| A/Heilongjiang-Yichun/1634/2016 | Asia / China / Heilongjiang | EPI817112 \| 2016-CX1846.4 | EPI_ISL_230738 |
| A/Gansu-Chengguan/1657/2016 | Asia / China / Gansu | EPI777976 \| 2016-CX1265.4 | EPI_ISL_225415 |
| A/Anhui-Luyang/1311/2016 | Asia / China / Anhui | EPI777975 \| 2016-CX1264.4 | EPI_ISL_225414 |
| A/Shanxi-Chengqu/1187/2016 | Asia / China / Shanxi | EPI777827 \| 2016-CX1513.4 | EPI_ISL_225364 |
| A/Shandong-Decheng/1333/2016 | Asia / China / Shandong | EPI777824 \| 2016-CX1511.4 | EPI_ISL_225363 |
| A/Jilin-Ningjiang/1222/2016 | Asia / China / Jilin | EPI777821 \| 2016-CX1510.4 | EPI_ISL_225362 |
| A/Shanghai-Pudongxin/1429/2016 | Asia / China / Shanghai | EPI777818 \| 2016-CX1509.4 | EPI_ISL_225361 |
| A/Neimenggu-Xilinhaote/1166/2016 | Asia / China / Neimenggu | EPI777815 \| 2016-CX1508.4 | EPI_ISL_225360 |
| A/Tianjin-Nankai/1405/2016 | Asia / China / Tianjin | EPI777812 \| 2016-CX1507.4 | EPI_ISL_225359 |
| A/Guangdong-Zhongshan/1215/2016 | Asia / China / Guangdong | EPI777809 \| 2016-CX1506.4 | EPI_ISL_225358 |
| A/Liaoning-Lianshan/1173/2016 | Asia / China / Liaoning | EPI777806 \| 2016-CX1505.4 | EPI_ISL_225357 |
| A/Beijing-Xicheng/1540/2016 | Asia / China / Beijing | EPI777803 \| 2016-CX1504.4 | EPI_ISL_225356 |
| A/Gansu-Jingyuan/317/2016 | Asia / China / Gansu | EPI777800 \| 2016-CX1503.4 | EPI_ISL_225355 |
| A/Shaanxi-Hantai/1357/2016 | Asia / China / Shaanxi | EPI777797 \| 2016-CX1502.4 | EPI_ISL_225354 |
| A/Hebei-Lunan/1410/2016 | Asia / China / Hebei | EPI777794 \| 2016-CX1501.4 | EPI_ISL_225353 |
| A/Ningxia-Zhongning/1175/2016 | Asia / China / Ningxia | EPI777791 \| 2016-CX1500.4 | EPI_ISL_225352 |
| A/Jiangxi-Yuehu/1299/2016 | Asia / China / Jiangxi | EPI777692 \| 2016-CX1266.4 | EPI_ISL_225319 |
| A/Shanghai-Pudongxin/1493/2016 | Asia / China / Shanghai | EPI777689 \| 2016-CX1263.4 | EPI_ISL_225318 |
| A/Heilongjiang-Longsha/1274/2016 | Asia / China / Heilongjiang | EPI777686 \| 2016-CX1262.4 | EPI_ISL_225317 |
| A/Zhejiang-Wuxin/1300/2016 | Asia / China / Zhejiang | EPI777683 \| 2016-CX1261.4 | EPI_ISL_225316 |
| A/Shandong-Laicheng/1104/2016 | Asia / China / Shandong | EPI777680 \| 2016-CX1259.4 | EPI_ISL_225315 |
| A/Jiangsu-Sucheng/1309/2016 | Asia / China / Jiangsu | EPI777677 \| 2016-CX1258.4 | EPI_ISL_225314 |
| A/Heilongjiang-Jianhua/142/2016 | Asia / China / Heilongjiang | EPI769342 \| 2016-CX0495.4 | EPI_ISL_223602 |
| A/Shaanxi-Beilin/135/2016 | Asia / China / Shaanxi | EPI769339 \| 2016-CX0496.4 | EPI_ISL_223601 |
| A/Shanghai-Pudongxin/141/2016 | Asia / China / Shanghai | EPI769336 \| 2016-CX0498.4 | EPI_ISL_223600 |
| A/Shanxi-Taigu/17/2016 | Asia / China / Shanxi | EPI769333 \| 2016-CX0499.4 | EPI_ISL_223599 |
| A/Shanxi-Yuci/124/2016 | Asia / China / Shanxi | EPI769330 \| 2016-CX0500.4 | EPI_ISL_223598 |
| A/Anhui-Xiangshan/16/2016 | Asia / China / Anhui | EPI769327 \| 2016-CX0501.4 | EPI_ISL_223597 |
| A/Heilongjiang-Longsha/118/2016 | Asia / China / Heilongjiang | EPI769324 \| 2016-CX0502.4 | EPI_ISL_223596 |
| A/Heilongjiang-Gongnong/112/2016 | Asia / China / Heilongjiang | EPI769321 \| 2016-CX0503.4 | EPI_ISL_223595 |
| A/Heilongjiang-Yichun/140/2016 | Asia / China / Heilongjiang | EPI769318 \| 2016-CX0504.4 | EPI_ISL_223594 |
| A/Heilongjiang-Aihui/14/2016 | Asia / China / Heilongjiang | EPI769315 \| 2016-CX0505.4 | EPI_ISL_223593 |
| A/Shandong-Taishan/11/2016 | Asia / China / Shandong | EPI769312 \| 2016-CX0506.4 | EPI_ISL_223592 |
| A/Shaanxi-Qindou/141/2016 | Asia / China / Shaanxi | EPI769309 \| 2016-CX0507.4 | EPI_ISL_223591 |
| A/Gansu-Chengguan/1127/2016 | Asia / China / Gansu | EPI769306 \| 2016-CX0508.4 | EPI_ISL_223590 |
| A/Zhejiang-Yongkang/147/2016 | Asia / China / Zhejiang | EPI769303 \| 2016-CX0509.4 | EPI_ISL_223589 |
| A/Zhejiang-Nanxun/138/2016 | Asia / China / Zhejiang | EPI769300 \| 2016-CX0512.4 | EPI_ISL_223588 |
| A/Gansu-Qinzhou/114/2016 | Asia / China / Gansu | EPI769297 \| 2016-CX0513.4 | EPI_ISL_223587 |
| A/Yunnan-Xishan/150/2016 | Asia / China / Yunnan | EPI769294 \| 2016-CX0514.4 | EPI_ISL_223586 |
| A/Yunnan-Lushui/153/2016 | Asia / China / Yunnan | EPI769291 \| 2016-CX0515.4 | EPI_ISL_223585 |
| A/Yunnan-Hongta/1180/2016 | Asia / China / Yunnan | EPI769288 \| 2016-CX0516.4 | EPI_ISL_223584 |
| A/Shanghai-Pudongxin/154/2016 | Asia / China / Shanghai | EPI769285 \| 2016-CX0517.4 | EPI_ISL_223583 |
| A/Gansu-Chengguan/523/2016 | Asia / China / Gansu | EPI769282 \| 2016-CX0518.4 | EPI_ISL_223582 |
| A/Shanxi-Taigu/160/2016 | Asia / China / Shanxi | EPI769279 \| 2016-CX0519.4 | EPI_ISL_223581 |
| A/Gansu-Jingyuan/137/2016 | Asia / China / Gansu | EPI769276 \| 2016-CX0520.4 | EPI_ISL_223580 |
| A/Shanxi-Yuci/147/2016 | Asia / China / Shanxi | EPI769273 \| 2016-CX0521.4 | EPI_ISL_223579 |
| A/Shanxi-Chengqu/140/2016 | Asia / China / Shanxi | EPI769270 \| 2016-CX0522.4 | EPI_ISL_223578 |
| A/Shanxi-Xinghualing/14/2016 | Asia / China / Shanxi | EPI769267 \| 2016-CX0523.4 | EPI_ISL_223577 |
| A/Heilongjiang-Hailin/120/2016 | Asia / China / Heilongjiang | EPI769264 \| 2016-CX0524.4 | EPI_ISL_223576 |
| A/Heilongjiang-Aimin/126/2016 | Asia / China / Heilongjiang | EPI769261 \| 2016-CX0525.4 | EPI_ISL_223575 |
| A/heilongJiangxi-An/114/2016 | Asia / China / heilongJiangxi | EPI769258 \| 2016-CX0526.4 | EPI_ISL_223574 |
| A/Heilongjiang-Daowai/171/2016 | Asia / China / Heilongjiang | EPI769255 \| 2016-CX0527.4 | EPI_ISL_223573 |
| A/Heilongjiang-Daoli/125/2016 | Asia / China / Heilongjiang | EPI769252 \| 2016-CX0528.4 | EPI_ISL_223572 |
| A/Heilongjiang-Nangang/161/2016 | Asia / China / Heilongjiang | EPI769249 \| 2016-CX0530.4 | EPI_ISL_223571 |
| A/Heilongjiang-Longsha/137/2016 | Asia / China / Heilongjiang | EPI769246 \| 2016-CX0531.4 | EPI_ISL_223570 |
| A/Heilongjiang-Aihui/121/2016 | Asia / China / Heilongjiang | EPI769243 \| 2016-CX0532.4 | EPI_ISL_223569 |
| A/Heilongjiang-Xiangyang/138/2016 | Asia / China / Heilongjiang | EPI769240 \| 2016-CX0534.4 | EPI_ISL_223568 |
| A/Zhejiang-Yongkang/111/2016 | Asia / China / Zhejiang | EPI769237 \| 2016-CX0535.4 | EPI_ISL_223567 |
| A/Heilongjiang-Longsha/145/2016 | Asia / China / Heilongjiang | EPI769234 \| 2016-CX0536.4 | EPI_ISL_223566 |
| A/Heilongjiang-Taoshan/124/2016 | Asia / China / Heilongjiang | EPI769231 \| 2016-CX0539.4 | EPI_ISL_223565 |
| A/Heilongjiang-Aihui/137/2016 | Asia / China / Heilongjiang | EPI769228 \| 2016-CX0540.4 | EPI_ISL_223564 |
| A/Liaoning-Shuangta/135/2016 | Asia / China / Liaoning | EPI769225 \| 2016-CX0541.4 | EPI_ISL_223563 |
| A/Liaoning-Yinzhou/112/2016 | Asia / China / Liaoning | EPI769222 \| 2016-CX0542.4 | EPI_ISL_223562 |
| A/Jilin-Dongchang/141/2016 | Asia / China / Jilin | EPI769219 \| 2016-CX0543.4 | EPI_ISL_223561 |
| A/Jilin-Tiexi/117/2016 | Asia / China / Jilin | EPI769216 \| 2016-CX0544.4 | EPI_ISL_223560 |
| A/Jilin-Chuanying/118/2016 | Asia / China / Jilin | EPI769213 \| 2016-CX0545.4 | EPI_ISL_223559 |
| A/Jilin-Chaoyang/162/2016 | Asia / China / Jilin | EPI769210 \| 2016-CX0546.4 | EPI_ISL_223558 |
| A/Jilin-Nanguan/16/2016 | Asia / China / Jilin | EPI769207 \| 2016-CX0547.4 | EPI_ISL_223557 |
| A/Guangxi-Xiangshan/143/2016 | Asia / China / Guangxi | EPI769204 \| 2016-CX0548.4 | EPI_ISL_223556 |
| A/Hebei-Xinhua/1124/2016 | Asia / China / Hebei | EPI769201 \| 2016-CX0549.4 | EPI_ISL_223555 |
| A/Hebei-Yuhua/148/2016 | Asia / China / Hebei | EPI769198 \| 2016-CX0550.4 | EPI_ISL_223554 |
| A/Hebei-Zunhua/15/2016 | Asia / China / Hebei | EPI769195 \| 2016-CX0551.4 | EPI_ISL_223553 |
| A/Hebei-Lunan/143/2016 | Asia / China / Hebei | EPI769192 \| 2016-CX0552.4 | EPI_ISL_223552 |
| A/Hebei-Yuhua/119/2016 | Asia / China / Hebei | EPI769189 \| 2016-CX0553.4 | EPI_ISL_223551 |
| A/Hebei-Changan/117/2016 | Asia / China / Hebei | EPI769186 \| 2016-CX0554.4 | EPI_ISL_223550 |
| A/tianjinHebei/117/2016 | Asia / China / tianjinHebei/117/2016 | EPI769183 \| 2016-CX0555.4 | EPI_ISL_223549 |
| A/Tianjin-Nankai/171/2016 | Asia / China / Tianjin | EPI769180 \| 2016-CX0556.4 | EPI_ISL_223548 |
| A/Tianjin-Beichen/112/2016 | Asia / China / Tianjin | EPI769177 \| 2016-CX0557.4 | EPI_ISL_223547 |
| A/Henan-Zhongzhan/39/2016 | Asia / China / Henan | EPI769174 \| 2016-CX0558.4 | EPI_ISL_223546 |
| A/Henan-Ruyang/120/2016 | Asia / China / Henan | EPI769171 \| 2016-CX0559.4 | EPI_ISL_223545 |
| A/Henan-Luolong/118/2016 | Asia / China / Henan | EPI769168 \| 2016-CX0560.4 | EPI_ISL_223544 |
| A/Henan-Xigong/125/2016 | Asia / China / Henan | EPI769165 \| 2016-CX0561.4 | EPI_ISL_223543 |
| A/Zhejiang-Yongkang/124/2016 | Asia / China / Zhejiang | EPI769162 \| 2016-CX0562.4 | EPI_ISL_223542 |
| A/Zhejiang-Nanxun/111/2016 | Asia / China / Zhejiang | EPI769159 \| 2016-CX0564.4 | EPI_ISL_223541 |
| A/Hunan-Tianyuan/18/2016 | Asia / China / Hunan | EPI769156 \| 2016-CX0565.4 | EPI_ISL_223540 |
| A/Shandong-Tianqiao/15/2016 | Asia / China / Shandong | EPI769153 \| 2016-CX0566.4 | EPI_ISL_223539 |
| A/Shaanxi-Qindou/134/2016 | Asia / China / Shaanxi | EPI769150 \| 2016-CX0409.4 | EPI_ISL_223538 |
| A/Yunnan-Xianggelila/137/2016 | Asia / China / Yunnan | EPI769147 \| 2016-CX0410.4 | EPI_ISL_223537 |
| A/Shanxi-Yaodou/1327/2016 | Asia / China / Shanxi | EPI769144 \| 2016-CX0411.4 | EPI_ISL_223536 |
| A/Zhejiang-Yiwu/198/2016 | Asia / China / Zhejiang | EPI769141 \| 2016-CX0412.4 | EPI_ISL_223535 |
| A/Jilin-Chaoyang/1121/2016 | Asia / China / Jilin | EPI769138 \| 2016-CX0414.4 | EPI_ISL_223534 |
| A/Neimenggu-Kundoulun/1140/2016 | Asia / China / Neimenggu | EPI769135 \| 2016-CX0417.4 | EPI_ISL_223533 |
| A/Shanxi-Lishi/126/2016 | Asia / China / Shanxi | EPI769132 \| 2016-CX0421.4 | EPI_ISL_223532 |
| A/Shanxi-Chengqu/174/2016 | Asia / China / Shanxi | EPI769129 \| 2016-CX0422.4 | EPI_ISL_223531 |
| A/Guangxi-Wanxiu/196/2016 | Asia / China / Guangxi | EPI769126 \| 2016-CX0423.4 | EPI_ISL_223530 |
| A/Shanxi-Yuci/1105/2016 | Asia / China / Shanxi | EPI769123 \| 2016-CX0424.4 | EPI_ISL_223529 |
| A/Shanxi-Taigu/1112/2016 | Asia / China / Shanxi | EPI769120 \| 2016-CX0425.4 | EPI_ISL_223528 |
| A/Zhejiang-Wuxin/165/2016 | Asia / China / Zhejiang | EPI769117 \| 2016-CX0426.4 | EPI_ISL_223527 |
| A/Jilin-Chaoyang/1137/2016 | Asia / China / Jilin | EPI769114 \| 2016-CX0430.4 | EPI_ISL_223526 |
| A/Jilin-Longshan/192/2016 | Asia / China / Jilin | EPI769111 \| 2016-CX0431.4 | EPI_ISL_223525 |
| A/Jilin-Dongchang/190/2016 | Asia / China / Jilin | EPI769108 \| 2016-CX0432.4 | EPI_ISL_223524 |
| A/Jilin-Hunjiang/1114/2016 | Asia / China / Jilin | EPI769105 \| 2016-CX0433.4 | EPI_ISL_223523 |
| A/Anhui-Xuanzhou/1134/2016 | Asia / China / Anhui | EPI769102 \| 2016-CX0434.4 | EPI_ISL_223522 |
| A/Neimenggu-Kundoulun/1186/2016 | Asia / China / Neimenggu | EPI769099 \| 2016-CX0435.4 | EPI_ISL_223521 |
| A/Heilongjiang-Taoshan/1132/2016 | Asia / China / Heilongjiang | EPI769096 \| 2016-CX0436.4 | EPI_ISL_223520 |
| A/Heilongjiang-Gongnong/1115/2016 | Asia / China / Heilongjiang | EPI769093 \| 2016-CX0441.4 | EPI_ISL_223519 |
| A/Shaanxi-Weicheng/13/2016 | Asia / China / Shaanxi | EPI769090 \| 2016-CX0443.4 | EPI_ISL_223518 |
| A/Heilongjiang-Daowai/163/2016 | Asia / China / Heilongjiang | EPI769087 \| 2016-CX0445.4 | EPI_ISL_223517 |
| A/Heilongjiang-Saertu/1102/2016 | Asia / China / Heilongjiang | EPI769084 \| 2016-CX0449.4 | EPI_ISL_223516 |
| A/Heilongjiang-Yichun/1143/2016 | Asia / China / Heilongjiang | EPI769081 \| 2016-CX0450.4 | EPI_ISL_223515 |
| A/Hebei-Ningjin/196/2016 | Asia / China / Hebei | EPI769078 \| 2016-CX0452.4 | EPI_ISL_223514 |
| A/Hebei-Hejian/1101/2016 | Asia / China / Hebei | EPI769075 \| 2016-CX0453.4 | EPI_ISL_223513 |
| A/Hebei-Xinhua/1103/2016 | Asia / China / Hebei | EPI769072 \| 2016-CX0454.4 | EPI_ISL_223512 |
| A/Hebei-Luquan/157/2016 | Asia / China / Hebei | EPI769069 \| 2016-CX0455.4 | EPI_ISL_223511 |
| A/Hebei-Jizhou/1121/2016 | Asia / China / Hebei | EPI769066 \| 2016-CX0456.4 | EPI_ISL_223510 |
| A/Hebei-Yuhua/1128/2016 | Asia / China / Hebei | EPI769063 \| 2016-CX0457.4 | EPI_ISL_223509 |
| A/Hebei-Lunan/1144/2016 | Asia / China / Hebei | EPI769060 \| 2016-CX0458.4 | EPI_ISL_223508 |
| A/Hebei-Zunhua/134/2016 | Asia / China / Hebei | EPI769057 \| 2016-CX0459.4 | EPI_ISL_223507 |
| A/Hebei-Yunhe/1123/2016 | Asia / China / Hebei | EPI769054 \| 2016-CX0460.4 | EPI_ISL_223506 |
| A/Hebei-Taocheng/196/2016 | Asia / China / Hebei | EPI769051 \| 2016-CX0461.4 | EPI_ISL_223505 |
| A/Hebei-Jizhou/188/2016 | Asia / China / Hebei | EPI769048 \| 2016-CX0462.4 | EPI_ISL_223504 |
| A/Neimenggu-Huimin/11027/2016 | Asia / China / Neimenggu | EPI769045 \| 2016-CX0463.4 | EPI_ISL_223503 |
| A/Neimenggu-Jining/139/2016 | Asia / China / Neimenggu | EPI769042 \| 2016-CX0464.4 | EPI_ISL_223502 |
| A/Heilongjiang-Daoli/158/2016 | Asia / China / Heilongjiang | EPI769039 \| 2016-CX0470.4 | EPI_ISL_223501 |
| A/Heilongjiang-Gongnong/149/2016 | Asia / China / Heilongjiang | EPI769036 \| 2016-CX0471.4 | EPI_ISL_223500 |
| A/Liaoning-Lianshan/158/2016 | Asia / China / Liaoning | EPI769033 \| 2016-CX0472.4 | EPI_ISL_223499 |
| A/Liaoning-Haizhou/153/2016 | Asia / China / Liaoning | EPI769030 \| 2016-CX0473.4 | EPI_ISL_223498 |
| A/Liaoning-Shuncheng/161/2016 | Asia / China / Liaoning | EPI769027 \| 2016-CX0474.4 | EPI_ISL_223497 |
| A/Liaoning-Mingshan/136/2016 | Asia / China / Liaoning | EPI769024 \| 2016-CX0475.4 | EPI_ISL_223496 |
| A/Liaoning-Donggang/116/2016 | Asia / China / Liaoning | EPI769021 \| 2016-CX0477.4 | EPI_ISL_223495 |
| A/Beijing-Huairou/1235/2016 | Asia / China / Beijing | EPI769018 \| 2016-CX0480.4 | EPI_ISL_223494 |
| A/Anhui-Yaohai/321/2016 | Asia / China / Anhui | EPI769015 \| 2016-CX0482.4 | EPI_ISL_223493 |
| A/Henan-Hubin/116/2016 | Asia / China / Henan | EPI769012 \| 2016-CX0483.4 | EPI_ISL_223492 |
| A/Jiangsu-Chongchuan/18/2016 | Asia / China / Jiangsu | EPI713670 \| 2016-CX0247.4 | EPI_ISL_212677 |
| A/Heilongjiang-Jianhua/15/2016 | Asia / China / Heilongjiang | EPI713589 \| 2016-CX0254.4 | EPI_ISL_212650 |
| A/Heilongjiang-Longsha/111/2016 | Asia / China / Heilongjiang | EPI713586 \| 2016-CX0255.4 | EPI_ISL_212649 |
| A/Shanxi-Xinghualing/121/2016 | Asia / China / Shanxi | EPI713571 \| 2016-CX0260.4 | EPI_ISL_212644 |
| A/Jiangsu-Haizhou/118/2016 | Asia / China / Jiangsu | EPI713565 \| 2016-CX0241.4 | EPI_ISL_212642 |
